# Sonochemical degradation of CCl_4_ and its effects on the degradation of organic pollutants in water: A review

**DOI:** 10.1016/j.ultsonch.2026.108050

**Published:** 2026-09-18

**Authors:** Kenji Okitsu, Aerfate Abulikemu

**Affiliations:** Graduate School of Sustainable System Sciences, Osaka Metropolitan University, 1-1 Gakuen-cho, Naka-ku, Sakai, Osaka 599-8531, Japan

**Keywords:** CCl_4_, Ultrasonic cavitation, Degradation, Dosimetry, Water treatment

## Abstract

•OH radicals formed from the sonolysis of water can be used for the degradation of various hazardous organic compounds. This water treatment technique is actively studied as an •OH-based advanced oxidation process. However, the sonochemical formation of •OH radicals requires a large amount of electrical energy so that this technique is facing the challenge of high costs. In this review, we focused on the sonlysis of CCl_4_, where the degradation rate of CCl_4_ is one to two orders of magnitude higher than that of water. Based on the comparison of the characteristics of CCl_4_ with related organic compounds, CCl_4_ was suggested to undergo the fastest sonochemical degradation among organic compounds. Weissler *et al*. proposed that •Cl radicals formed from the sonolysis of CCl_4_ in water act as effective oxidizing agents for I^−^. This reaction is known as the Weissler reaction and has been used to evaluate chemical effects of ultrasonic cavitation. In addition, •Cl radicals are effective in accelerating the degradation rate of various hazardous organic compounds such as dyes, aromatics, antibiotics, etc. in water. Since CCl_4_ can also undergo reductive degradation, the reduction of CCl_4_ is of interest for environmental remediation. To review the knowledge on the sonolysis of CCl_4_, a rapidly degradable organic compound, would be meaningful for the future development of a high efficient ultrasonic water treatment technique.

## 1 Introduction

Sonochemical degradation has been investigated as a treatment technique for water containing hazardous organic pollutants. When ultrasound is applied to an aqueous solution, cavitation bubbles are generated, and their adiabatic compression collapse can produce extremely high temperatures and pressures inside the bubbles. Three reaction sites are generated by the collapse of the bubbles in water. (I) Inside the bubble: temperatures exceeding several thousand degrees and pressures exceeding several hundred atmospheres transiently exist [1], [2]. Water molecules and volatile organic compounds present inside the bubble are thermally degraded. For example, •OH and •H radicals are formed by the thermal homolytic cleavage of water molecules [3]. (II) Interfacial region of the bubble: the average temperature in this region is also at a high level [2] so that the thermal degradation of organic compounds present here occurs. Furthermore, the concentration of •OH radicals is high in this region so that organic compounds can be rapidly degraded [4]. (III) Bulk solution: this region is at ambient temperature and pressure. Unreacted radicals escaping from region (II) react with organic compounds present in region (III). Therefore, the sonochemical degradation of organic compounds in water undergoes direct pyrolysis reactions in region (I) and (II) and/or •OH radical reactions in region (II) and (III). In addition, physical effects such as shock waves, microjets, acoustic streaming, and intense micro- and macro-mixing are generated in the solution during ultrasonic cavitation [1], [2].

•OH radicals formed during ultrasonic cavitation can be used for the degradation and detoxification of various hazardous organic compounds so that this ultrasonic cavitation technique is known as •OH-based advanced oxidation processes (AOPs) [5], [6]. This ultrasonic technique is also considered as an environmentally friendly one due to the main advantages including low secondary pollution, low requirement for additional oxidants or other chemicals, and synergistic effects with other technologies. However, because the sonochemical formation of •OH radicals requires a large amount of electrical energy [7], the practical application of the sonochemical degradation technique is limited by its high cost. Therefore, the improvement of the degradation efficiency has become one of the most important issues.

This review will focus on the sonochemical degradation of CCl_4_, because CCl_4_ is a more easily degradable compound than water. This is because the bond dissociation energy of Cl–CCl_3_ (293 kJ mol^−1^) is lower than that of H–OH (498.7 kJ mol^−1^) [8]. In addition, the bond dissociation energies of Cl–CCl_3_ and Cl–C (338 kJ mol^−1^) [8] are lower than those of representative chemical bonds. For example, the following bond energies are reported: H–CH_3_ (431 kJ mol^−1^) [8], H_3_C–CH_3_ (377 kJ mol^−1^) [9], HO–CH_3_ (377 kJ mol^−1^) [8], and H–OCH_3_ (437.6 kJ mol^−1^) [9]. On the other hand, the bond dissociation energies of Br–CBr_3_ (209 kJ mol^−1^) [8] and Br–C (280 kJ mol^−1^) [8] are lower than those of Cl–CCl_3_ and C–Cl [8]. However, the pyrolysis of CBr_4_ is difficult to occur because most of CBr_4_ molecules could not be present inside the bubble due to its low vapor pressure (for example, 5.33 kPa at 96°C [10], there was no reliable data at around room temperature). In other words, the moderate vapor pressure (12.2 kPa at 20°C [11]) of CCl_4_ makes it more likely to be present inside the bubble. The characteristics of these chemicals and related chemicals are shown in Table 1. It is probable that CCl_4_ undergoes thermal degradation very readily among them. The Henry's law constants of the chemicals [12] are also listed in Table 1, because those data are important parameters for evaluating the progress of reactions inside the bubbles in water. The details are described in the later section.

**Table 1:** Characteristics of CCl_4_, related chemicals, methanol, and water.

	CCl_4_, 153.82 ^a)^	CHCl_3_, 119.37 ^a)^	CH_2_Cl_2_, 84.93 ^a)^	CBr_4_, 331.63 ^a)^	CH_4_, 16.043 ^a)^	CH_3_OH, 32.04 ^a)^	H_2_O, 18.015 ^a)^
Bond dissociation energy / kJ mol^−1^	Cl–CCl_3_; 293 ^8)^	H–CCl_3_; 377 ^8)^	H–CHCl_2_; 414.2 ^8)^ Cl–CH_2_Cl; 310 ^8)^	Br–CBr_3_; 209 ^8)^	H–CH_3_; 431 ^8)^	HO–CH_3_; 377 ^8)^ H–OCH_3_; 436.8 ^9)^	H–OH; 498.7 ^8)^
Solubility in water ^10)^ / mM	5.2 (20 ℃)	67 (20 ℃)	153 (20 ℃)	0.72 (30 ℃)	1.37 (25 ℃, 1 atm)	Miscible	—
Log *P*^10)^	2.83	1.97	1.25	3.42	1.09	−0.77	—
Vapor pressure / kPa	12.2 (20 °C) ^10)^	21.2 (20 ℃) ^10)^	47.4 (20 ℃) ^10)^	5.33 (96 °C) ^10)^	—	12.9 (20 °C) ^10)^	2.34 (20 ℃)^11)^
Henry's law constant at 25 °C ^12)^ / Pa m^3^ mol^−1^	2900	370	260	—	71,400	0.5	—
Boiling point ^10)^ / ℃	76.7	61.12	39.75	189.5	−161.50	64.7	100

a) Molecular weight.

“—“: Not available.

Besides, CCl_4_ is a structurally simple compound, containing only C–Cl bonds, which makes it convenient to compare the results of its sonolysis with those of water. In this paper, we review how CCl_4_ degradation proceeds very fast in the research fields of (1) I^–^ oxidation in the presence of CCl_4_, (2) degradation of CCl_4_, (3) degradation of hazardous organic compounds in the presence of CCl_4_, and (4) reductive degradation of CCl_4_.

In the introduction section, an outline of previous research on the sonochemical degradation of CCl_4_ is briefly summarized. First, Weissler *et al*. [13] studied on the formation of I_3_^–^ from the sonolysis of an aqueous KI solution containing CCl_4_. This reaction, known as the “Weissler reaction”, is widely used as a dosimeter to evaluate the performance of sonochemical reactors and reaction system. In the Weissler reaction, the yield of I_3_^–^ formed is monitored by a UV–Vis spectrophotometer, one of the most widely used instruments, so that the analysis of this reaction is convenient in the laboratory. In addition, since the yields of •Cl radicals and related oxidants formed from the sonolysis of CCl_4_ are larger than those of •OH radicals and H_2_O_2_ formed from the sonolysis of water, the Weissler reaction is often useful for analyzing cavitation with low chemical activity.

On the other hand, the sonolysis of CCl_4_ has been studied from the viewpoint of environmental conservation. For example, it has been reported that the initial rate of degradation of CCl_4_ is 6.6 to 36 times faster than that of water. Furthermore, the addition of CCl_4_ accelerates the sonochemical degradation rates or increases the degradation rate constants of co-existing organic compounds (dyes, aromatics, antibiotics, etc.) by 1.4- to 160-fold. The reductive dechlorination of CCl_4_ also proceeds readily. Understanding the sonochemical degradation of CCl_4_ will contribute to the development of efficient water treatment technologies in the future.

## 2 Weissler reaction

When bubbles with high-temperatures and high-pressures are formed, water and/or organic compounds present inside the bubbles are thermally degraded. According to the research by Weissler *et al*. [13], when ultrasonic irradiation is applied to an aqueous KI solution containing CCl_4_, CCl_4_ is thermally degraded to form •Cl radicals and the following reactions proceed:

(1) •Cl + I^−^ → Cl^−^ + •I

(2) 2•Cl → Cl_2_

(3) 2•I → I_2_

(4) Cl_2_ + 2I^−^ → 2Cl^−^ + I_2_

(5) I^−^ + I_2_ → I_3_^−^

The amount of •Cl radicals and Cl_2_ can be evaluated spectroscopically from the amount of I_3_⁻ formed, where the molar absorption coefficient for I_3_⁻ is 26,000 M^−1^ cm^−1^ at 352 nm. They also reported the progress of the following reactions:

(6) CCl_4_ + H_2_O → CO + 2HCl + Cl_2_

(7) 2HCl + [O] → Cl_2_ + H_2_O

where [O] is oxidants such as H_2_O_2_.

In addition, since four •Cl radicals are formed from the degradation of one CCl_4_ molecule [13], the molar amount of I_3_^−^ formed would correspond to half of the molar amount of CCl_4_ degradation. It is possible that the reactions such as Cl_2_ + H_2_O → HCl + HClO, Cl_2_ + H_2_O_2_ → 2HCl + O_2_, and HClO + H_2_O_2_ → O_2_ + H_2_O + HCl occur. However, these reactions are complex and unclear, therefore, we will not discuss them quantitatively here. •Cl + Cl^−^ → •Cl_2_^−^ also occur and •Cl_2_^−^ is an oxidant. Weissler *et al*. also investigated systematically the effects of various parameters on I^−^ oxidation in the presence of CCl_4_ such as test-tube diameter, wall thickness of test-tube, distance between test-tube and crystal oscillator, solution temperature, solution volume, KI concentration, CCl_4_ volume, ultrasound frequency, ultrasound input power, and gas type.

On the other hand, the sonolysis of aqueous KI solutions under Ar in the absence of CCl_4_ forms •OH radicals, H_2_O_2_, and H_2_, where •OH radicals and H_2_O_2_ act as oxidants for I^−^ as follows:

(8) H_2_O → •OH +^.^H

(9) 2•OH → H_2_O_2_

(10) 2•H → H_2_

(11) •OH + I^−^ → OH^−^ + •I

(12) H_2_O_2_ + 2I^−^ → 2OH^−^ + I_2_

The amount of •OH radicals and H_2_O_2_ can be also evaluated by the amount of I_3_^−^ formed.

Weissler *et al*. reported that the rates of I_3_^–^ formation in the presence of CCl_4_ was several to several tens of times faster than those in the absence of CCl_4_ [13]. In other words, the degradation of CCl_4_ proceeded one to two orders of magnitude faster than that of water. Weissler *et al*. used various amounts of CCl_4_ [13]. In some cases, the added CCl_4_ was too high to soluble in water perfectly. The solubility of CCl_4_ in water is 5.2 mM at 20°C [10]. When a large amount of CCl_4_ was added in an aqueous solution, part of the added CCl_4_ (specific gravity: 1.59 g cm^−3^ at 20°C) should deposit at the bottom of the solution before ultrasonic irradiation. In such a case, the reactions proceed heterogeneously or emulsification may be induced by ultrasonic irradiation. If so, the reactions would be very complicated.

Fig. 1 shows a high-temperature and high-pressure bubble formed in water and the outline of three reaction sites. As shown in Fig. 1, the sonolysis of an organic compound is achieved effectively when a larger amount of organic compound molecules is present at the interface region and inside of the bubble. On the other hand, it should be noted that if the large amount of CCl_4_ molecules was present inside the bubble, the bubble temperature after adiabatic compression would not reach a high temperature due to the small specific heat ratio of CCl_4_ (specific heat ratio (γ) = 1.13) [14]. The details are described in Section 3.

**Fig. 1:**
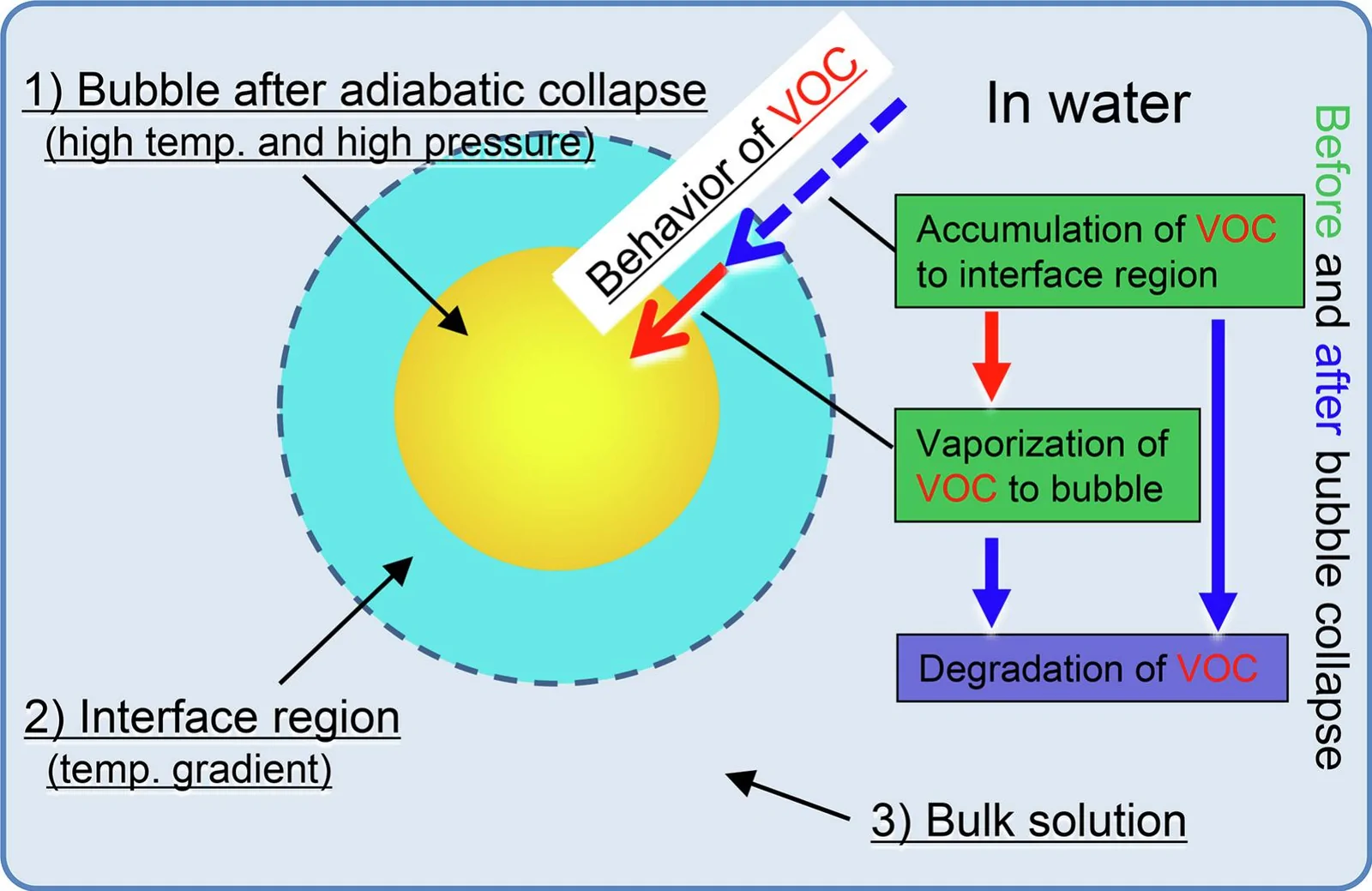
Three reaction sites in water. (1) Inside bubble, (2) interface region, and (3) bulk solution. Before bubble collapse, accumulation of a volatile organic compound molecule (VOC) occurs from region (3) to region (2) and vaporization of VOC and water occurs from region (2) to region (1).

It is probable that Henry's law constant is used as an indicator when we consider the progress of reactions inside the bubble in water. Henry’s law equation is expressed as follows:

H=P/C

where *H* is the partial pressure of the organic compound in the gas phase (Pa), *C* is the concentration of the organic compound dissolved in water (mol L^−1^), and *H* is the Henry’s law constant (Pa m^3^ mol^−1^). Hung and Hoffmann investigated the effect of the Henry’s law constant on the sonochemical degradation of volatile chlorinated methane at 205 kHz in water [15]. Fig. 2 shows the pseudo-first-order kinetics plots obtained from the sonolysis of CCl_4_, CHCl_3_, and CH_2_Cl_2_ [15]. It can be seen that the order of the degradation rate was CH_2_Cl_2_ < CHCl_3_ < CCl_4_ and this order was consistent with the order of Henry's law constant. The data of *H* are listed in Table 1. Since compounds with a high *H* value tend to be more volatile from aqueous solution, those that more readily volatilize into bubbles would degrade more rapidly [15]. However, when referring to the value of Henry's law constant, it may be necessary to take into account the time corresponding to the number of bubble oscillations, where the number of oscillations is equivalent to the bubble lifetime. Sunartio *et al*. reported that the number of oscillations was estimated to be 70–110 when 213, 358, 647, and 1062 kHz ultrasound were used [16]. If the number of bubble oscillations was assumed to be 100 times at 205 kHz, the corresponding time could be calculated to be ca. 0.5 ms. The timescale of 0.5 ms may not be sufficient for an organic compound to reach evaporation equilibrium between the liquid phase and the gas phase inside the bubble. For such a short time, it would be important to consider both of the effect of Henry's law constant and vaporization rate of organic compounds: equilibrium theory and kinetics must be considered on a case-by-case basis. On the other hand, the fact that the bond dissociation energy of Cl–CCl_3_ (293 kJ mol^−1^) [8] is lower than those of H–CCl_3_ (377 kJ mol^−1^) [8], H–CHCl_2_ (414.2 kJ mol^−1^) [8], and Cl–CH_2_Cl (310 kJ mol^−1^) [8] could also affect the rates of degradation of CCl_4_, CHCl_3_, and CH_2_Cl_2_.

**Fig. 2:**
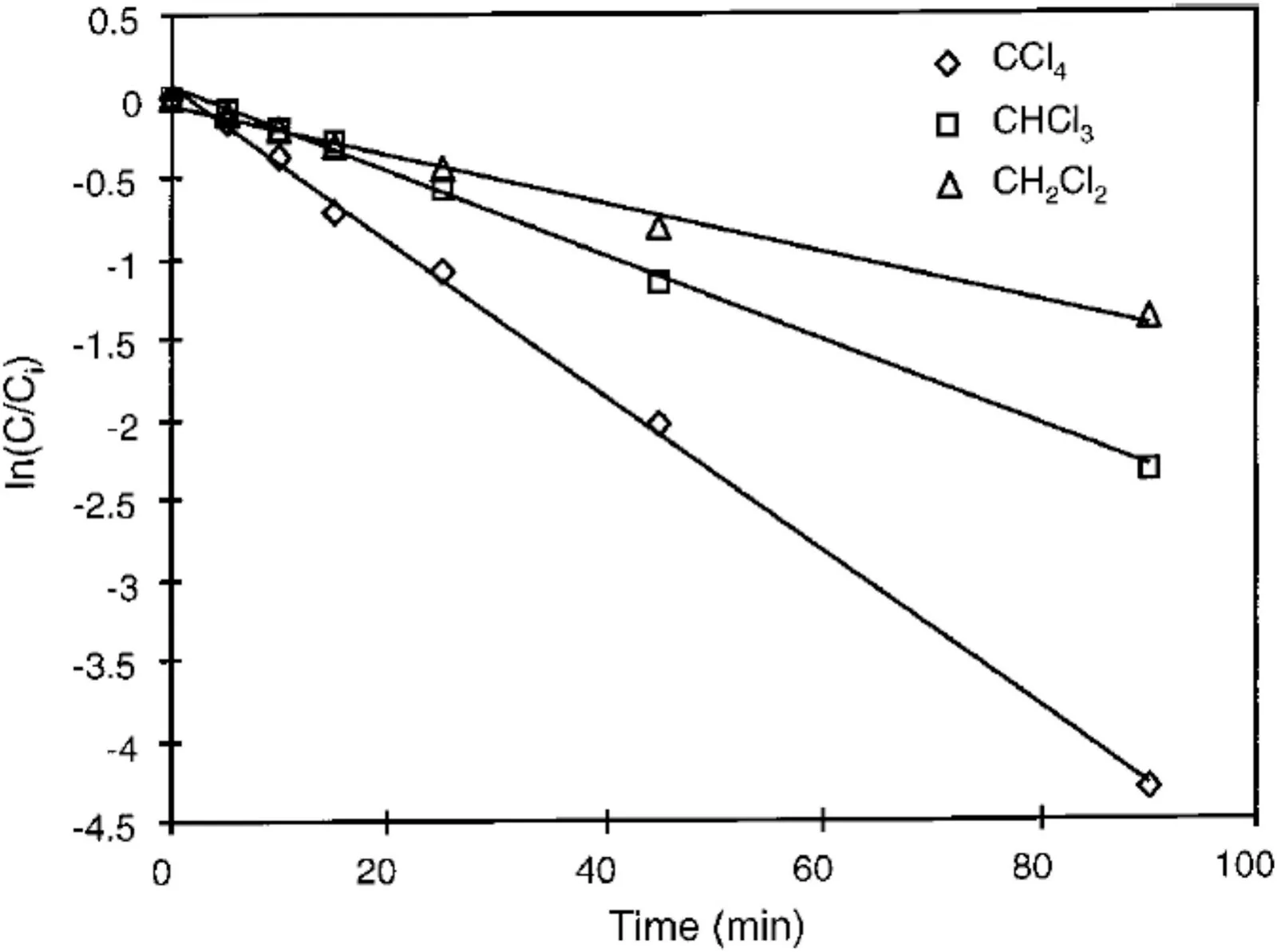
Observed first-order kinetic plots for the degradation of CCl_4_, CHCl_3_, and CH_2_Cl_2_ at 205 kHz [15]. Copyright, 1999 ACS.

Since I_3_⁻ is efficiently formed from the sonolysis of an aqueous CCl_4_-KI solution, the amount of I_3_^−^ formed has been used as a dosimetry to evaluate the chemical effects of ultrasonic cavitation or ultrasonic experimental systems. Here, we briefly introduce representative dosimetry methods (electron spin resonance (ESR) method, Fricke dosimetry, terephthalic acid method, and sonoluminescence method) for the evaluation of the formation of •OH radicals and/or related oxidants such as H_2_O_2_. Chemical dosimetry methods are also compared and discussed in detail in references [17], [18].

ESR method: The concentration of radicals can be measured using an ESR apparatus. In general, it is difficult to directly measure the amount of •OH radicals formed because of their short lifetime. Therefore, •OH radicals are converted into long-lived radical species for analysis. For example, when 5,5-dimethyl-1-pyrroline-N-oxide (DMPO), a spin-trapping agent, is added to a solution for ultrasonic irradiation, the •OH radicals formed are trapped by DMPO and converted into long-lived DMPO–OH radicals. The DMPO–OH radicals can then be measured using an ESR apparatus to estimate the amount of •OH radicals formed [19]. This method requires relatively expensive apparatus and reagents.

Fricke dosimetry: The Fricke dosimetry, originally used in the field of radiation chemistry, can be used to measure the amounts of •OH radicals and H_2_O_2_ formed in a solution. In the experiment, the sample solution including Fe^2+^ and H_2_SO_4_ is sonicated and then the amount of Fe^3+^ formed is analyzed using a UV–visible spectrophotometer [20], [21]. The molar absorption coefficient for Fe^3+^ is 2194 M^−1^ cm^−1^ at λ = 304 nm. The molar absorption coefficient for Fe^3+^ is an order of magnitude smaller than that for I_3_^−^ in the KI method. Therefore, the KI method may be more suitable for analyzing low concentrations of oxidants.

Terephthalic acid method: The sample solution containing terephthalic acid (non-fluorescent) is sonicated, resulting in the formation of 2-hydroxyterephthalic acid (fluorescent, HTA). HTA is excited at 315  nm and fluorescence intensity of HTA is measured at 425  nm by a fluorometer. The limit of detection for •OH radicals is reported to be about 1.3 × 10^−9^ M [22]. For example, since this method enables highly sensitive analysis, this method can be used to analyze the chemical effects of single bubble cavitation [23].

Sonoluminescence method: The amount of •OH radicals formed is evaluated by analyzing the light emitted from excited •OH radicals within the high-temperature and high-pressure cavitation bubbles. In general, the emission originating from •OH radicals is observed at around 310 nm [24], [25], [26]. As a related approach, sonochemiluminescence is used, where an alkaline aqueous solution containing luminol is sonicated and then the luminescence from luminol oxidation is analyzed [26]. Photographs of sonoluminescence and sonochemiluminescence can be used to visualize the spatial distribution of the reaction field.

In this section, we introduce the dosimetry using I^−^ oxidation. Table 2 shows the representative results of I^−^ oxidation in the presence of CCl_4_ [13], [27], [28], [29], [30], [31], where both results of the Weissler method and KI method are listed. Recently, although the KI method in the absence of CCl_4_ is also referred to as the Weissler method [17], [32], we refer to the original Weissler method using CCl_4_ simply as “the Weissler method” in this paper. This definition could be also supported by the recent review paper [18]. In addition, in Table 2, we distinguish between the Weissler method and the KI method as follows: in the Weissler method, KI reagent was added to the sample solution in the presence of CCl_4_ before ultrasonic irradiation, but in the KI method presented here, KI reagent was added to the sample solution in the presence of CCl_4_ after ultrasonic irradiation. When these terms were defined above, there appear to be no reports after 2016 as shown in Table 2. In this table, the sonicator types were also categorized as disk, bath, and horn. In addition, I_3_^−^ yield, irradiation time, initial oxidation rate, and rate constant were used to evaluate the enhancement of I⁻ oxidation by the addition of CCl_4_. In notes, the referred Table number or Figure number in the original paper are shown. The result using the KI method defined here is also shown in Notes. It can be seen in Table 2 that the amount of I_3_^−^ formed or the rate of I_3_^−^ formation are 1.4–37 times larger in the presence of CCl_4_ than in the absence of CCl_4_, where the result of Alippi *et al*. was not included in this enhancement calculation because the data were not shown clearly in their paper. Ghodbane and Hamdaoui investigated the effect of the concentration of CCl_4_ on the I_3_^–^ formation rate at 1700 kHz ultrasound [29]. It was confirmed that the I_3_^–^ formation rate increased from 12.5 to 37.2 times as the concentration of CCl_4_ increased from 0.650 to 5.19 mM. Since the size, size distribution, number, and life time of bubbles and their bubble dynamics are affected by ultrasonic frequency and power, the effects of ultrasonic frequency and power are still important research subjects to be clarified in sonochemistry.

**Table 2:** Sonolysis of aqueous CCl_4_ solution. Enhancement of I⁻ oxidation.

	Sonicator type, Water temp.	Ultrasound frequency	Ultrasound power ^a)^	Dissolved gas	Solution volume	CCl_4_ conc. ^b)^	KI conc.	Rate of I⁻ oxidation ^c)^	Enhancement	Notes (without CCl_4_)
[13] Weissler *et al*. (1950)	Disk, ―	1000 kHz	310 W	O_2_ N_2_air	100.5 mL	0.5 mL (52 mM)	1 M	20.5 × 10^-5^ equiv. time: 5 min	24 times 15 times14 times	(1.5 × 10^-5^ equiv.) Table 2
[27] Alippi *et al*. (1992)	Bath, ―	―	300 W	air	240 mL	2.38 g (64.5 mM)	4.5 M	8.9 × 10^-4^ M time: 3 h	three orders of magnitude	(no data without CCl_4_) Table 2
[28] Zhou *et al*. (2007).	Horn, 11 ± 2℃	20 kHzpulsed mode (2 s on/1s off)	3.2 W	―	5 mL	20 mg/L (0.13 mM)	20 mM	(Not reported) time: 12 min	5 times	Enhancement was calculated from the data points shown in Fig. 2
[29] Ghodbane and Hamdaoui (2009)	Disk, 20 ± 1℃	1700 kHz	14 W: cal.	―	100 mL	100 mg L^−1^ (0.650 mM) 160 mg L^−1^ (1.04 mM) 239 mg L^−1^ (1.55 mM) 399 mg L^−1^ (2.59 mM) 798 mg L^−1^ (5.19 mM)	0.1 M	12.96 μM min^−1^ 18.42 μM min^−1^ 27.68 μM min^−1^ 35.76 μM min^−1^ 38.64 μM min^−1^	12.5 times 17.7 times 26.6 times 34.4 times37.2 times	(1.04 μM min^−1^) Fig. 4
[30] Gogate and Katekhaye (2012)	Horn, 30 ± 2℃	20 kHz	29.3 W: cal.	―	300 mL	0.2 wt% (13 mM)	300 ppm	(Not reported) time: 60 min	2.65 times	Fig. 6
[30] Gogate and Katekhaye (2012)	Bath, ―	204 kHz	52.4 W	―	3300 mL	0.2 wt% (13 mM)	300 ppm	(Not reported) time: 60 min	1.4 times	Fig. 6
[31] Uddin and Okitsu (2016)	Disk, ―	200 kHz	16 W: cal.	Ar	60 mL	8.6 mM	0.4 M	(Not reported) time: 5 min	6.2 times	KI method Fig. 5

In Notes, the referred Table number or Figure number in the original paper are shown.

Results using KI method are also shown in Notes.

“—“: Not available.

a) Calorimetric power was abbreviated as cal. No description means nominal electric power.

b) Values in parentheses were converted to the unit of mM.

c) Yield at irradiation time (abbreviate as time), initial oxidation rate (abbreviate as initial rate, μM min^−1^), or rate constant.

d) Not available.

Shock tube techniques have long been used to study high-temperature reactions and the pyrolysis of water [33] and CCl_4_ [34] has been investigated, respectively. The activation energies for eqs. (14), (15) were reported to be 392 kJ mol^−1^ [33] in the temperature range of 2196–3290 K and 183 kJ mol^−1^ [34] in the range of 1470–2000 K, respectively:

(14) H_2_O + M → •H + •OH + M

(15) CCl_4_ + M → •CCl_3_ + •Cl + M

where M represents a third body.

Considering the activation energies for Eqs. (14), (15) and bond dissociation energies of Cl–CCl_3_ and H–OH, the threshold temperature at which the homolytic cleavage occurs should be lower for Cl–CCl_3_ than H–OH (Fig. 3). This idea suggests that the degradation of CCl_4_ could proceed even in the low-temperature bubbles where the sonolysis of water does not occur. Such situation should occur after adiabatic compression collapse when a large amount of CCl_4_ molecules is present inside the bubble, where the specific heat ratio (γ) of CCl_4_ is smaller than those of dissolved gases (ex. Ar, air, etc.) and water vapor. This idea is also suggested by the fact that the Weissler reaction has been used to evaluate the chemical effect of hydrodynamic cavitation, which produces less H_2_O_2_ than ultrasonic cavitation [35], [36], [37], [38]. In recent years, Dehane *et al*. reported simulations on the temperature dependence of chemical reactions occurring in argon single bubble cavitation in aqueous CCl_4_ solutions [39]. Fig. 4 shows the temperature dependence of the rate constants of the reactions: Fig. 4a (kf1 and kr1) shows H_2_O + M ⇌ H + OH + M, Fig. 4b (k20 and kr20) CCl_4_ ⇌ CCl_3_ + Cl, Fig. 4c shows (k28 and kr28) CCl_2_ + H ⇌ HCl + CCl, and Fig. 4d shows (k29 and kr29) Cl + H_2_O ⇌ OH + HCl, respectively, as a function of bubble temperature. From the results of Fig. 4a and b, it was clear that the rate constant of forward reaction of kf20 (sonolysis of CCl_4_) is higher than that of kf1 (sonolysis of water).

**Fig. 3:**
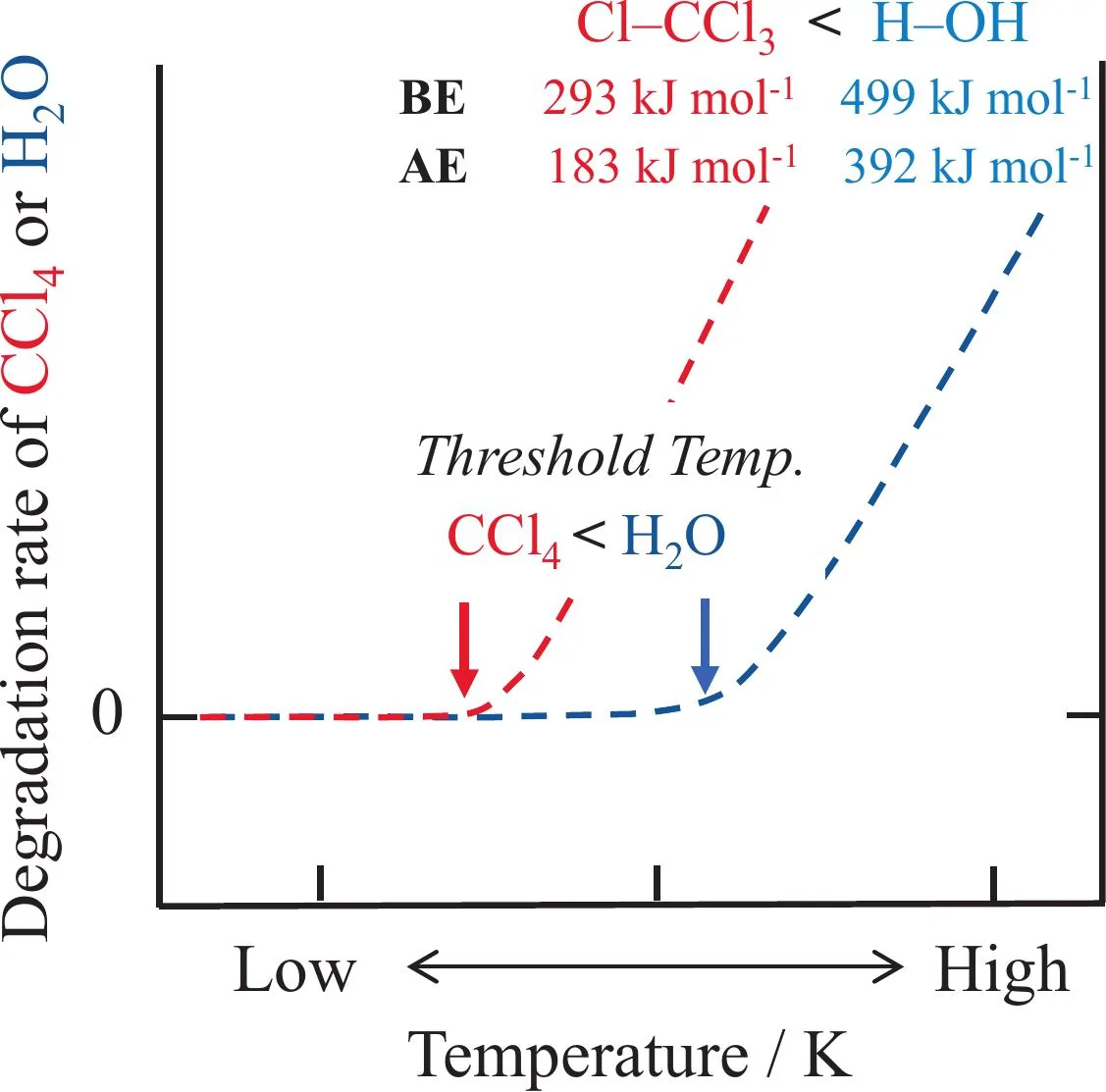
A schematic illustration of thermal degradation of CCl_4_ and H_2_O. CCl_4_ could undergo degradation even in low-temperature bubbles where the degradation of water does not occur. This idea is suggested both by the bond dissociation energy (BE) and by the activation energy (AE) of these chemicals estimated by the data of shock tube techniques.

**Fig. 4:**
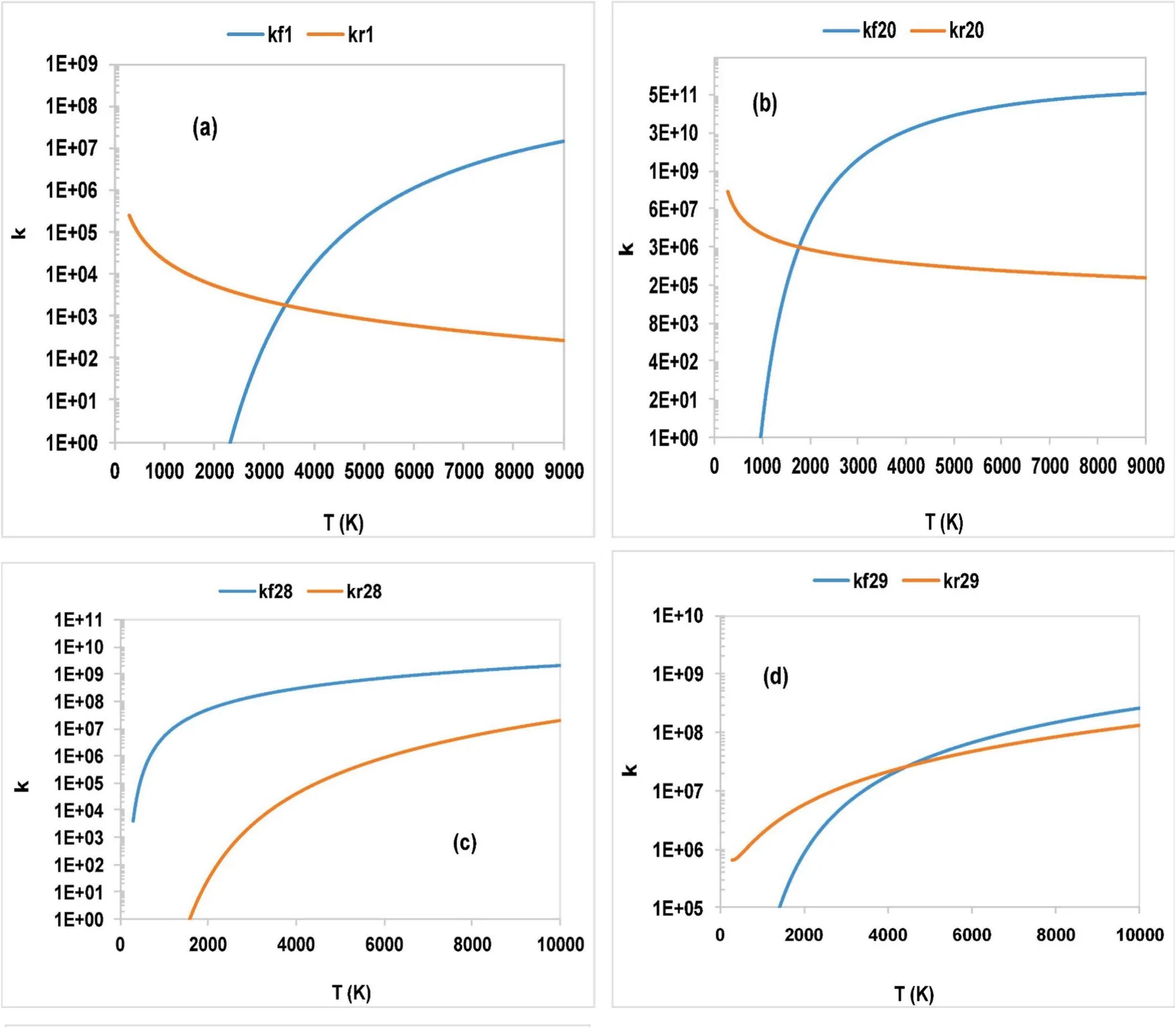
Evolutions vs. temperature of the rate constants of reactions (kf1 and kr1) H_2_O + M ⇌ H + OH + M, (k20 and kr20) CCl_4_ ⇌ CCl_3_ + Cl, (k28 and kr28) CCl_4_ + H ⇌ HCl + CCl_3_, and (k29 and kr29) Cl + H_2_O ⇌ OH + HCl, where “f” denotes the forward reaction and “r” denotes the reverse reaction. Unit: (m^3^ mol^−1^ s^−1^) for two body reaction and (m^6^ mol^−2^ s^−1^) for a three body reaction. Vertical axes are in logarithmic scale. (Reprinted with permission from Ref. [39]. Copyright 2021 Elsevier).

At present, the causal relationship between the bubble temperature and the degradation rate of molecules inside the bubble is still unclear experimentally. This is because the temperature, pressure, reaction time, and reaction space change complexly during ultrasonic cavitation. Therefore, further studies are needed.

## 3 Sonochemical degradation of CCl_4_ in water

CCl_4_ is categorized as a volatile organic compound (VOC) and has been used as a raw material for refrigerants, cleaning solvents, etc. However, its use is severely restricted due to its hazardous properties. Nevertheless, CCl_4_ is still widely discharged in the world, and there is concern about CCl_4_ pollution [40], [41]. Therefore, the development of degradation technique of CCl_4_ is an important issue from the viewpoint of environmental conservation. Due to the hazardous properties of VOCs such as trichloroethylene, tetrachloroethylene, 1,1,1-trichloroethylene, etc., the sonochemical degradation of VOCs has been studied. The sonochemical degradation of chlorofluorocarbons and hydrochlorofluorocarbons, which have similar chemical structures of chlorinated hydrocarbons, has also been studied. For these studies, refer to the cited references [42], [43], [44]. Dehane et al. reported simulations of bubble temperature and the sonochemistry of CCl_4_ in aqueous solutions [39], [45]. They analyzed the cavitation of a single argon bubble, and the radial dynamics of the bubble were calculated using the modified Keller–Miksis equation. They also simulated the yields of chemical species, including chlorine species (•CCl_3_, **:**CCl_2_, •Cl, and HOCl) and •OH, as a function of the CCl_4_ mole fraction. Their results showed that increasing the CCl_4_ mole fraction inside the bubble decreased the maximum bubble temperature, whereas the yields of the chemical species tended to increase with increasing CCl_4_ mole fraction. Additional information on their simulations can be found in the cited references [39], [45].

There are several early reports on the sonochemical degradation of CCl_4_ in aqueous solutions around the 1950s–1960s. For example, Jennings and Townsend reported the sonochemical degradation of CCl_4_ in aqueous solutions under argon from the viewpoint of the analysis of the degradation mechanism of CCl_4_ [46]. In their experiments, ultrasound was applied to a two-phase solution consisting of 20 mL of water and 2 mL of CCl_4_. It is difficult to interpret the results within the framework of aqueous sonochemistry, because emulsification would occur under such a condition. In this section, we summarized papers published since the 1990s, when studies began to focus on environmental conservation.

Table 3 shows the results of the sonochemical degradation of CCl_4_ in water [15], [47], [48], [49], [50], [51], [52], [53], [54], [55], [56], [59], [60]. Cheung *et al*. were the first to investigate the degradation of CCl_4_ from the viewpoint of degradation of environmental pollutants [47]. They investigated the degradation of CCl_4_ (1.04 mM) using a 20 kHz horn-type ultrasonic generator. Inazu *et al*. investigated the degradation of CCl_4_ (0.065 mM) under air using a 200 kHz standing-wave-type ultrasonic generator [48]. They suggested that the pyrolysis of CCl_4_ occurred directly in high-temperature and high-pressure bubbles. On the other hand, Nagata *et al*. [49] investigated the degradation of a high concentration of CCl_4_ (3.25 mM) using the same irradiation system as Inazu *et al*. Surprisingly, it was confirmed that the degradation rate of CCl_4_ was 36 times faster than that of water. As seen in Table 3, the results comparing between the degradation rate of CCl_4_ and formation rate of •OH (corresponding to the sonolysis rate of water) are very limited, but it is clear that the degradation of CCl_4_ occurs very fast. Petrier and Francony investigated the effect of ultrasonic frequency on the sonolysis of water and CCl_4_ [53]. The frequency dependence of the sonolysis of water was relatively clear, as the maximum rate was observed at 500 kHz, but the frequency dependence of the degradation of CCl_4_ was different from that of water. On the other hand, Hung and Hoffmann reported that the rate of the degradation of CCl_4_ was maximum at 500 kHz [15]. This result suggested that the chemical efficiency of ultrasonic cavitation may be maximum at around 500 kHz. This result was in good agreement with that of Koda *et al*. They used seven types of sonochemical apparatus operating at frequencies ranging from 19.5 kHz to 1.2 MHz for experiments with aqueous solutions in the absence of CCl_4_ and indicated that the highest sonochemical efficiency for I_3_^−^ formation was in the range of 200–500 kHz [57]. Later, it was also reported that the liquid height or solution volume is important, when the effect of ultrasonic frequency is discussed [58].

**Table 3:** Rate of CCl_4_ degradation in the sonolysis of aqueous solution and rate of OH formation in the sonolysis of pure water.

	Sonicator type, Water temp.	Ultrasound frequency	Ultrasound power ^a)^	Dissolved gas	Solution volume	CCl_4_ conc.^b)^	Rate of CCl_4_ degradation ^c)^	Comparison with rate of OH formation^g)^	Notes
[47] Cheung *et al*. (1991)	Horn, 15-20℃	20 kHz	250 W	―	―	100 ppmv (1.0 mM)	―	―	
[48] Inazu *et al*. (1992)	Disk, ―	200 kHz	200 W	air	65 mL	10 ppm (0.065 mM)	16.3 μM min^−1^ Initial rate ^d)^	―	Fig. 1
[49] Nagata *et al*. (1993)	Disk, ―	200 kHz	200 W	Ar	65 mL	500 ppm (3.3 mM)	710 μM min^−1^ Initial rate ^d)^	36 times	h): 9.9 μM min^−1^ i): 19.8 μM min^−1^ Fig. 1
[50] Bhatnagar and Cheung (1994)	Horn, 20-25℃	20 kHz	200 W	―	2000 mL	61 mg L^−1^ (0.40 mM)	0.043 min^−1^	―	Fig. 4
[51] Hua and Hoffmann (1996)	Horn, 25℃	20 kHz	135 W	Ar	95 mL	0.0195 mM 0.195 mM	3.9 × 10^−3^ s^−1^ 3.3 × 10^−3^ s^−1^	―	Fig. 2
[52] Francony and Petrier (1996)	Horn, 25℃	20 kHz	30 W: cal.	―	200 mL	3.5 mM	88 μM min^−1^ Initial rate ^e)^	―	Fig. 1
[52] Francony and Petrier (1996)	Disk, 25℃	500 kHz	30 W: cal.	―	200 mL	3.5 mM	220 μM min^−1^ Initial rate ^f)^	―	Fig. 1
[53] Petrier and Francony (1997)	Horn, 20℃	20 kHz	30 W: cal	O_2_	300 mL	0.4 mM	19 μM min^−1^	27 times	h): 0.7 μM min^−1^ i): 0.7 μM min^−1^ Table 1
[53] Petrier and Francony (1997)	Disk, 20℃	200 kHz 500 kHz 800 kHz	30 W: cal	O_2_	300 mL	0.4 mM	33 μM min^−1^ 37 μM min^−1^ 50 μM min^−1^	6.6 times 18 times36 times	h): 5, 2.1, and 1.4 μM min^−1^ i): 5, 2.1, 1.4 μM min^−1^ Table 1
[15] Hung and Hoffmann (1999)	Horn, 286 K	20 kHz	62 W: cal.	―	95 mL	0.2 mM	0.025 min^−1^	―	Table 3
[15] Hung and Hoffmann (1999)	Disk, 286 K	205 kHz 358 kHz 500 kHz 618 kHz 1078 kHz	35 W: cal. 35 W: cal. 48 W: cal. 35 W: cal. 35 W: cal.	― ― ― ― ―	605 mL 605 mL 483 mL 605 mL 605 mL	0.2 mM	0.044 min^−1^ 0.049 min^−1^ 0.070 min^−1^ 0.055 min^−1^ 0.039 min^−1^	―	Table 3
[54] Colussi *et al*. (1999)	Disk, 283 K	205 kHz 358 kHz 618 kHz 1078 kHz	50 W	Ar	605 mL	0.15 mM	7.3 × 10^-4^ s^−1^ 8.2 × 10^-4^ s^−1^ 9.2 × 10^-4^ s^−1^ 6.5 × 10^-4^ s^−1^	―	Table 1
[55] Lim *et al*. (2007)	Horn, 10 ℃ 20 ℃40 ℃	20 kHz	1 W mL^−1^	air	350 mL	100 mg L^−1^ (0.65 mM)	0.0286 min^−1^ 0.0233 min^−1^ 0.0197 min^−1^	―	Table 1
[56] Lee and Oh (2010)	Horn, 24 ℃	20 kHz	34 W: cal.	air	270 mL	1 mg L^−1^ (0.0065 mM) 5 mg L^−1^ (0.033 mM) 10 mg L^−1^ (0.065 mM)	0.0536 min^−1^ 0.0553 min^−1^ 0.0527 min^−1^	―	Table 1
[59] Lim et al. (2011)	Disk, 16 ± 2 ℃	35 kHz 74 kHz 170 kHz 300 kHz 1000 kHz	calorimetric power (18.8 ± 2.2 W)	―	―	0.05 mM	0.005 min^−1^ 0.003 min^−1^ 0.002 min^−1^ 0.011 min^−1^ 0.006 min^−1^	―	Fig. 4
[60] Abulikemu and Okitsu (2025)	Disk, 20 °C	200 kHz	13.6 W: cal.	Ar	100 mL	1.035 mM (1-propanol: 14.7 mM)	134 μM min^−1^	―	Fig. 2

In Notes, the referred Table number or Figure number in the original paper are shown.

“—“: Not available.

a) Calorimetric power was abbreviated as cal. No description means nominal electric power.

b) Values in parentheses were converted to the unit of mM.

c) Irradiation time (abbreviate as time), initial degradation rate (abbreviate as initial rate, μM min^−1^), or rate constant.

d) Data point at 2 min in Fig. 1 was used for rate estimation.

e) Data point at 10 min in Fig. 1 was used for rate estimation.

f) Data point at 4 min in Fig. 1 was used for rate estimation.

g) CCl_4_ degradation rate comparison with OH formation rate in the sonolysis of pure water.

h) H_2_O_2_ formation rate.

i) Estimated OH formation rate.

In the sonochemical degradation of CCl_4_ and VOCs (trichloroethylene, tetrachloroethylene, 1,1,1-trichloroethylene, chloroform) under argon [48], CO and Cl^−^ were the main degradation products and CO_2_ was a minor product. In addition, the sonolysis of CCl_4_ occurs during a single cavitation event. This result was considered to be based on the formation behavior of products as a function of sonication time. This phenomenon is also confirmed by Hua and Hoffmann [51].

Hua and Hoffmann investigated the kinetics and mechanism of the sonolysis of CCl_4_ in water [51]. A representative mechanism for the sonolysis of CCl_4_ in water was proposed as follows [15], [51]:

(16) CCl_4_ → •Cl + •CCl_3_

(17) CCl_4_ → Cl_2_ +**:**CCl_2_

(18) •CCl_3_ → •Cl +**:**CCl_2_

(19) •CCl_3_ + •CCl_3_ → CCl_4_ +**:**CCl_2_

(20) •CCl_3_ + •CCl_3_ → C_2_Cl_6_

(21) **:**CCl_2_ +**:**CCl_2_ → C_2_Cl_4_

(22) **:**CCl_2_ + H_2_O → 2 HCl + CO

(23) 2 •Cl → Cl_2_

(24) Cl_2_ + H_2_O → HOCl + HCl

where a gas chromatograph-electron capture detector (GC-ECD) and a mass spectroscopy were used to identify the degradation products. Eqs. (16), (17) show the formation of •Cl, •CCl_3,_ Cl_2_, and**:**CCl_2_, where**:**CCl_2_ is dichlorocarbene. The formation of**:**CCl_2_ was confirmed as intermediate by utilizing the reaction with 2,3-dimethlybutene to form 1,1,-dichloro-2,2,3,3-tetramethylcyclopropane. Taking into account the formation of C_2_Cl_4_ (3.1 × 10^−3^ μM), C_2_Cl_6_ (8.4 × 10^-2^ μM), Cl^−^ (1100 μM), and HOCl (130 μM) at 90 min sonolysis of 400 μM of CCl_4_, it was confirmed that most of the CCl_4_ molecules was degraded to inorganic chemicals, and the amounts of intermediates identified were small [51].

In sonochemistry, it should be noted that the temperature of the bubble (refer to here as bubble temperature) during adiabatic compression collapse depends on the gas property and gas composition inside the bubble. To increase the bubble temperature, it is effective to use a gas with a high specific heat ratio (γ) based on the following equation [61].

(25) $$T_{\text{max}}=T_{0}{\left[R_{0}/R_{\text{min}}\right]}^{\text{3}(\gamma -1)}$$

where *T*_max_ is the maximum bubble temperature, *T*_0_ is the solution temperature, *R*_0_ is the bubble radius before collapse, and *R*_min_ is the bubble radius just after collapse.

In aqueous sonochemistry, gaseous products such as CO and CO_2_ are formed from the sonolysis of organic compounds [48]. In general, air and argon are often used as dissolved gases in irradiation experiments. In the presence of O_2_, CO oxidation to CO_2_ tends to proceed. If a certain amount of gaseous products with a lower γ value than the dissolved gases entered subsequently formed or nearby cavitation bubbles, those bubble temperatures would not rise sufficiently during adiabatic compression collapse, and thus the chemical effects of the bubble would be reduced [62], [63].

Based on this idea, the rapid degradation of CCl_4_ observed at its high concentration may be an unexpected result. Because CCl_4_ is highly volatile, a considerable amount of CCl_4_ molecules may enter the bubble during rectifying diffusion until the bubble grows to its critical size. An increase in the proportion of CCl_4_ inside the bubble should quench the bubble temperature during adiabatic compression collapse, because the γ value of CCl_4_ is much smaller than that of argon and air (argon: γ = 1.67, air: γ = 1.40 at 15°C and 1 atm, CCl_4_: γ = 1.13 at 20°C and 0.1 atm) [64]. This phenomenon can be also inferred from the fact that the bubble temperature decreased due to an increase in the proportion of water vapor (γ = 1.324 at 100 °C and 1 atm) [65] inside the bubble when the solution temperature was increased [62], [63], [65]. Nevertheless, the rapid degradation of CCl_4_ was observed at a high concentration of CCl_4_, suggesting that the degradation of CCl_4_ could occur at a lower temperature than that of water, as mentioned in Fig. 3.

In aqueous sonochemistry, it is important to note that the amount of organic compounds present at the interface region of the bubble varies depending on its hydrophobicity and surface excess property. For example, in an aqueous methanol solution at concentrations of a few mM or lower, only a very small number of methanol molecules would be present inside the bubble even though methanol has a higher vapor pressure than water [66], [67], [68]. Here, we compared the behavior of CCl_4_ in aqueous solution with that of methanol. At 20°C, the vapor pressures of CCl_4_ and methanol are close to each other, as 12.2 kPa and 12.9 kPa [10], respectively. For log P, where P is the octanol–water partition coefficient, CCl_4_ is 2.83 and methanol is −0.770 [10], indicating that CCl_4_ is more hydrophobic than methanol. As a result, in an aqueous solution, CCl_4_ is more likely to be present at the interfacial region of the bubble than methanol, and thus a larger amount of CCl_4_ would tend to vaporize into the bubble during rectifying diffusion. In addition, since the interaction between CCl_4_ and H_2_O is weaker than the interaction of hydrogen bond between methanol and H_2_O, CCl_4_ should be more easily volatilized from the aqueous solution than methanol. These ideas can be supported by the comparison of the magnitudes of Henry's law constants of CCl_4_ and methanol as shown in Table 1. It should be noted that the above phenomenon is the case of an aqueous solution in which the concentration of the organic compound is low. The vaporization rate of the organic compound is also an important factor, when the bubble lifetime is very short.

Furthermore, when analyzing the H_2_O_2_ formation rate in the sonolysis of pure water, the following reactions must be considered: when an aqueous solution was sonicated in the presence of O_2_ (e.g. air), •OOH radicals are formed via the reaction of •H atoms with O_2_, resulting in the formation of H_2_O_2_ as Eq. (27) [21].

(26) •H + O_2_ → •OOH

(27) 2 •OOH → H_2_O_2_ + O_2_

Taking into account Eqs. (25), (26), half of the H_2_O_2_ formation rate in the presence of O_2_ would be attributed to the reaction of H atoms with O_2_. In Table 3, this correction was performed for the results of Petrier and Francony [53].

Regarding the reaction kinetics for the sonochemical degradation of CCl_4_, a steady-state approximation for the amount of degradation products or intermediates has been performed [15]. Hung and Hoffmann confirmed that more than 99 % of CCl_4_ was degraded when 0.20 mM of CCl_4_ was sonicated for 90 min. The amounts of C_2_Cl_6_ (maximum concentration: about 80 nM) and C_2_Cl_4_ (maximum concentration: about 25 nM) formed were measured as degradation products by using a GC-ECD. They performed a kinetic analysis using the steady-state approximation for the amounts of •CCl_3_ and •Cl radicals formed as intermediates, where the analysis was based on the assumption that the degradation of CCl_4_ occurs homogeneously throughout the solution. Taking into account the actual behavior of CCl_4_ molecules toward bubbles, the degradation of CCl_4_ would not occur homogeneously in solution. In fact, the existence of bubbles in the solution should cause the heterogeneity of the CCl_4_ concentration among the inside of bubbles, the interfacial region of bubbles, and the bulk solution. To analyze the kinetics of the degradation occurring in bubbles, the accurate information for the CCl_4_ concentration in bubbles is necessary. In future studies, the details of kinetic analyses that account for the behavior of both bubbles and reactants in the solution should be elucidated. In addition, the size and number of bubbles reaching high temperatures and pressures, as well as the temperature and pressure at which the reactions occur, should be clarified.

From 2012 to 2024, there appear to be no reports on the sonochemical degradation of CCl_4_. Only limited research groups have been investigating the degradation of CCl_4_ for general parameters, such as the effects of ultrasound frequency, ultrasound power, and CCl_4_ concentration [15], [47], [48], [49], [50], [51], [52], [53], [54], [55], [56], [59]. Therefore, it may be still unclear how easily CCl_4_ can be degraded compared with water. Recently, it was reported that the degradation rate of CCl_4_ could be enhanced by using alcohol-CCl_4_ pretreatment solutions [60]. The addition of alcohol is a unique idea, because it is generally reported that the addition of alcohol mainly has negative effects on the sonochemical degradation of organic compounds, such as decreasing the bubble temperature, and quenching the oxidative degradation as an •OH radical scavenger. However, it was indicated that the addition of alcohol accelerated the rate of the sonochemical degradation of CCl_4_. The homogeneity of CCl_4_ in aqueous solution provided by an alcohol-CCl_4_ pretreatment solution was considered as one of the main reasons of the enhancement of the degradation of CCl_4_. In addition, it was suggested that the formation of •H radicals could contribute to the reductive degradation of CCl_4_. The detailed mechanism should be clarified in the future.

## 4 Effect of CCl_4_ addition on degradation of dyes

In many cases in aqueous sonochemistry, the degradation rate of a target organic compound which is not pyrolyzed could not exceed the formation rate of •OH radicals. In other words, the formation rate of •OH radicals could be the upper rate of their degradation in an aqueous solution. To enhance the degradation rate of the organic compound, it may be necessary to consider reactions other than •OH radical reactions. In the sonolysis of CCl_4_, it is reported that chlorine-containing radicals (•Cl, •CCl_3_, and**:**CCl_2_) are formed and they are often used as oxidants for the degradation of various organic compound as described later. Taking into account the small yield of C_2_Cl_6_ and C_2_Cl_4_ [51], •CCl_3_ and**:**CCl_2_ could be considered minor oxidants. Here, the effects of •Cl radicals and the related oxidants formed from the sonolysis of CCl_4_ and water on the degradation of dyes are introduced.

The addition of CCl_4_ to an aqueous solution containing a dye accelerates the degradation rate of the dye during ultrasonic irradiation. Wang *et al*. [69] and Okitsu *et al*. [70] reported the effect of CCl_4_ addition on the sonochemical degradation of methyl orange (MO), where MO was used as a model compound of azo dyes. Wang *et al*. sonicated an aqueous solution of MO (initial concentration: 10 mg L^−1^, solution volume: 5 mL) by using a 20 kHz horn-type ultrasonic generator. It was confirmed that the degradation rate constant of MO in the presence of 49.2 mg L^−1^ CCl_4_ increased to 0.635 min^−1^ that was 160 times faster than that in the absence of CCl_4_ [69]. Okitsu *et al*. conducted experiments using an ultrasonic cleaning bath (solution volume: 1500 mL, frequency: 45 kHz, nominal electric power: 100 W) and a standing-wave-type ultrasonic generator (disk type, solution volume: 60 mL, frequency: 200 kHz, nominal electric power: 200 W), respectively. In the standing-wave-type system, it was confirmed that the degradation ratio of MO (initial concentration: about 30 μM, irradiation time: 10 s) increased to 54 % that was about 100 times faster when 150 ppm CCl_4_ was included [70]. Even using an ultrasonic cleaning bath operating at low ultrasonic power, the degradation rate of MO was enhanced by the addition of CCl_4_: the degradation ratio increased from 4.8 to 14 times as the concentration of CCl_4_ increased from 100 to 250 ppm.

Zheng *et al*. reported that CCl_4_ acts as a •H radical scavenger and thus the reaction of (29) cannot proceed when •H radicals are not present. Since CCl_4_ does not react with •OH radicals, the yield of •OH radicals could increase compared with that in the absence of CCl_4_ [71].

(28) •H + CCl_4_ → •ClCl_3_ + HCl

(29) •H + •OH → H_2_O

(30) •OH + CCl_4_ → no reaction

According to this mechanism, MO in the presence of CCl_4_ should react with •OH radicals effectively. However, this reaction alone cannot account for the more than 100-fold enhancement in MO degradation reported in [69], [70]. Two reaction pathways via •OH radicals or •Cl radicals could be depending on the concentration of CCl_4_.

Table 4 summarizes the effects of CCl_4_ on the sonochemical degradation of various dyes [29], [31], [69], [70], [72], [73], [74], [75], [76], [77], [78]. The addition of CCl_4_ increased the degradation rate and degradation rate constant of malachite green [72], C.I. Acid Orange 8 [73], Acid Blue 25 [29], Rhodamine B [74], CI. Direct Yellow 9 [75], and Reactive brilliant red K-2BP (K-2BP) [76], methylene blue [77], and Safranin O [78], where the rates and degradation rate constants were enhanced by 3.8–160 times. The reasons for the remarkable enhancements could be considered to be the low initial concentration of the dye used in the experiment and the high reactivity of •Cl radicals and •OH radicals to the dye.

**Table 4:** Effect of CCl_4_ on sonochemical degradation of dye.

	Target chemical,Conc.	Sonicator type, Water temp.	Ultrasound frequency	Ultrasound power ^a)^	Dissolved gas, pH	Solution volume	CCl_4_ conc.^b)^	Rate of dye degradation with CCl_4_^c)^	Enhancement	Notes (Rate without CCl_4_)
[69] Wang *et al*. (2007)	Methyl orange 10 mg L^−1^	Horn14℃	20 kHz pulsed mode (2 s on 1 s off)	3.2 W: cal.	air, pH 6.4	5 mL	49.2 mg L^−1^ (0.32 mM)	0.635 min^−1^	160 times	(0.004 min^−1^) Fig. 2
[70] Okitsu *et al*. (2008)	Methyl orange 30 μM	Bath20 ℃	45 kHz	100 W	air, pH 2	1500 mL	100 ppm (0.65 mM) 150 ppm (0.98 mM) 200 ppm (1.3 mM) 250 ppm (1.6 mM)	8.7 % 16 % 19 % 26 %Degradation ratio	4.8 times 8.9 times 11 times 14 times	(1.8 %) Table 1
[70] Okitsu *et al*. (2008)	Methyl orange 30 μM	Disk20 ℃	200 kHz	200 W	air, pH 2	60 mL	50 ppm (0.33 mM) 100 ppm (0.65 mM) 150 ppm (0.98 mM)	4.0 % 22 % 54 %Degradation ratio	7.4 times 41 times 100 times	(0.54 %) Table 1
[72] Bejarano-Pérez and Suárez-Herrera (2008)	Malachite green 27.5 μM	Horn25 ℃	20 kHz	14 W	air	1000 mL	1 mL L^−1^ (10 mM)	(Not reported) Rate constant	20 times	Enhancement was calculated from the data points shown in Fig. 5
[73] Gültekin *et al*. (2009)	C.I. Acid Orange 8 30 μM	Disk20 ℃	300 kHz	25 W: cal.	air	100 mL	2.07 mM	0.2451 min^−1^	3.8 times	(0.0648 min^−1^) Table 2 and Fig. 2
[29] Ghodbane and Hamdaoui (2009)	Acid Blue 25 50 mg L^−1^	Disk20 ℃	1700 kHz	14 W: cal.	―	100 mL	399 mg L^−1^ (2.59 mM) 798 mg L^−1^ (5.19 mM)	15.609 mg L^−1^ min^−1^ 17.325 mg L^−1^ min^−1^	106 times 118 times	(0.1467 mg L^−1^ min^−1^) Fig. 3
[29] Ghodbane and Hamdaoui (2009)	Acid Blue 25 50 mg L^−1^	Horn20 ℃	22.5 kHz	14 W: cal.	―	100 mL	399 mg L^−1^ (2.59 mM)	3.6555 mg L^−1^ min^−1^	87 times	(0.0418 mg L^−1^ min^−1^) Fig. 7
[74] Merouani (2010)	Rhodamine B, 5 mg L^−1^	Disk25 ℃	300 kHz	60 W	–	300 mL	50 mg L^−1^ (0.33 mM) 100 mg L^−1^ (0.65 mM) 200 mg L^−1^ (0.98 mM)	(Not reported)Initial rate	2.4 times 4.6 times 21 times	Fig. 10
[75] Eren and Ince (2010)	CI. Direct Yellow 9 28.75 μ M	Disk20 ℃	577 kHz 861 kHz 1145 kHz	0.23 W mL^−1^	–	250 mL cell size	2.0 m M	55.91 × 10^−4^ s^−1^ 141.22 × 10^−4^ s^−1^ 106.09 × 10^−4^ s^−1^	38 times 146 times 80 times	(1.47 × 10^−4^ s^−1^) Table 2
[75] Eren and Ince (2010)	CI Reactive Red 141 28.75 μM	Disk20 ℃	577 kHz 861 kHz 1145 kHz	0.23 W mL^−1^	–	250 mL cell size	2.0 mM	32.56 × 10^−4^ s^−1^ 33.02 × 10^−4^ s^−1^ 34.41 × 10^−4^ s^−1^	10 times 9.0 times 9.1 times	(3.18 × 10^−4^ s^−1^) Table 2
[76] Wang *et al*. (2011)	Reactive brilliant red K-2BP 20 mg L^−1^	Horn20 ℃	20 kHzduty cycle: 50%	17.3 W: cal.	air	50 mL	0.04 mL (8.3 mM)	9.51 × 10^2^ min^−1^	25 times	(0.372 × 10^2^ min^−1^) Table 1
[31] Uddin and Okitsu (2016)	Methyl orange Methylene blue 150 μM	disk10 ℃	200 kHz	200 W 16 W: cal	Ar	60 mL	2.6 mM	(Not reported) Initial rate	MO (9.6times) MB (1.4times)	Fig. 7
[77] Li et al. (2021)	Methylene blue 40 mg / L	Horn30 ℃	20 kHz	1000 W	―, pH 1.95	200 mL	0.5 %	0.0918 min^−1^	5.8 times	(0.0159 min^−1^) Fig. 5
[78] Hamida *et al*. (2022)	Safranin O 10 mg L^−1^	Disk25℃	1.7 MHz	15 W: cal.	Ar, pH 7	100 mL	2 mM	5.41 µM min^−1^	14 times	(0.378 µM min^−1^) Fig. 1b)

In Notes, the referred Table number or Figure number in the original paper are shown.

“—“: Not available.

a) Calorimetric power was abbreviated as cal. No description means nominal electric power.

b) Values in parentheses were converted to the unit of mM.

c) Irradiation time (abbreviate as time), initial degradation rate (abbreviate as initial rate, μM min^−1^ or mg L^−1^ min^−1^), degradation ratio, or rate constant.

d) Estimated OH formation rate.

Next, we consider the rate constant of the reaction of •Cl radicals or •OH radicals with a dye. After literature survey, it was confirmed that the information for the rate constant of the reaction of •Cl radicals with a dye is limited and only methylene blue was reported [79]. Therefore, for comparison, the data for the representative or related organic compounds in this paper are shown in Table 5 [79], [80], [81], [82], [83], [84], [85]. It can be seen in Table 5 that the reaction of organic compounds with •Cl or •OH radicals proceeds with rate constants greater than 10^9^ mol^−1^ dm^3^ s^−1^, therefore, the reactions with •Cl radicals can be considered as a diffusion-controlled reaction. According to Wojnárovits *et al.* [80], the reactions of •Cl radical with organic molecules are similar to the •OH radical reactions, but •Cl radicals appears to be less selective. A key difference is that single-electron-transfer reactions are less important for •OH than for •Cl.

**Table 5:** Rate constant of the reaction of Cl or OH with representative chemicals [79], [80], [81], [82], [83], [84], [85].

Chemical	k_Cl_, mol^−1^ dm^3^ s^−1^ (Reference)	K_OH_, mol^−1^ dm^3^ s^−1^ (Reference)
Methylene blue	2.8 × 10^9^ (Hoang *et al*. 2022)	1.1 × 10^10^ (Ohno *et al*. 1971)
Methanol	9.0 × 10^9^ (Lei *et al*. 2019)	9.7 × 10^8 a)^ (Buxton *et al*. 1988)
Ethanol	(2.2 ± 0.3) × 10^9^ (Wicktor *et al*. 2003)	1.9 × 10^9 a)^ (Buxton *et al*. 1988)
1-propanol	(2.2 ± 0.4) × 10^9^ (Wicktor *et al*. 2003)	2.8 × 10^9b)^ (Buxton *et al*. 1988)
2-propanol	(3.2 ± 0.7) × 10^9^ (Wicktor *et al*. 2003)	1.9 × 10^9 a)^ (Buxton *et al*. 1988)
Benzene	(0.60–1.2) × 10^10^ (Alegre *et al*. 2000)	7.8 × 10^9c)^ (Buxton *et al*. 1988)
Benzoic acid	(1.35 ± 0.15) × 10^10^ (Lei *et al*. 2019)	5.9 × 10^9 a)^ (Buxton *et al*. 1988)
Aniline	(2.74 ± 0.31) × 10^10^ (Lei *et al*. 2019)	1.4 × 10^10 d)^ (Buxton *et al*. 1988)
Phenol	(1.12 ± 0.09) × 10^10^ (Lei *et al*. 2019)	1.3 × 10^10b)^ (Buxton *et al*. 1988)
Ciprofloxacin	(1.39 ± 0.35) × 10^10^ (Lei *et al*. 2019)	(2.15 ± 0.10) × 10^10^ (An *et al*. 2010)

a) Selected value, b) average of 3 values, c) average of 4 values, d) average of 5 values.

Merouani *et al*. [74] investigated that the sonochemical degradation of Rhodamine B in the presence and absence of CCl_4_ under the condition at 300 mL solution, 300 kHz, and 60 W of ultrasound. It was found that the initial degradation rate became 2.4 times larger by the addition of 50 mgL^−1^ of CCl_4_, 4.6 times larger by the addition of 100 mgL^−1^ of CCl_4_, and 21 times larger by the addition of 200 mgL^−1^ of CCl_4_, respectively. This is because the sonolysis of CCl_4_ occurs more effectively at its higher concentration.

Hamida *et al*. [78] reported the role of reactive chlorine species and OH radicals for the degradation of Safranin O in the presence of CCl_4_ at 1.7 MHz and 15 W. The effects of pH were investigated and it was confirmed that the highest degradation rate was observed at pH 7.0 under argon among pH 3, 7, 11, and 13. It was also found that the degradation proceeded faster at 20 °C than 45°C. The amounts of oxidants (reactive chlorine species and reactive oxygen species) produced via the sonolysis of CCl_4_ and H_2_O were the key parameters for Safranin O degradation with respect to the medium pH.

Wang *et al*. [76] identified the degradation intermediates and products of K-2BP by combining a gas chromatograph with a mass spectrometer (GC–MS) and an ethylic ester derivative method. The anions of K-2BP degradation products, such as oxalic acid, maleic acid, nitrate, and sulfate ions, were detected using an ion chromatograph. The main degradation products were confirmed as follows: 2,4-(phenyldiazenyl) phthalic acid, 1-isocyanatonaphthalene, phthalic acid, 1-chloro-2-isocyanatobenzene, benzoquinone, phenol, 6-chloro-1,3,5-triazine-2,4-diol, 2-chlorophenol, maleic acid, oxalic acid, nitrate, and sulfate ion. Based on these identified products, a possible degradation pathway of the dye K-2BP was proposed as shown in Fig. 5. The amounts of intermediates and products were not measured. Some chlorinated intermediates were observed in figure, but it was unclear whether these are originated from K-2BP or the products from chlorination of K-2BP, because K-2BP itself contains chlorine atoms in both an aromatic ring and an s-triazine ring, respectively. At present, the report for sonochemical degradation mechanism of dyes in the presence of CCl_4_ is very limited. The degradation mechanism of ciprofloxacin (antibiotic) is also discussed in Section 5.

**Fig. 5:**
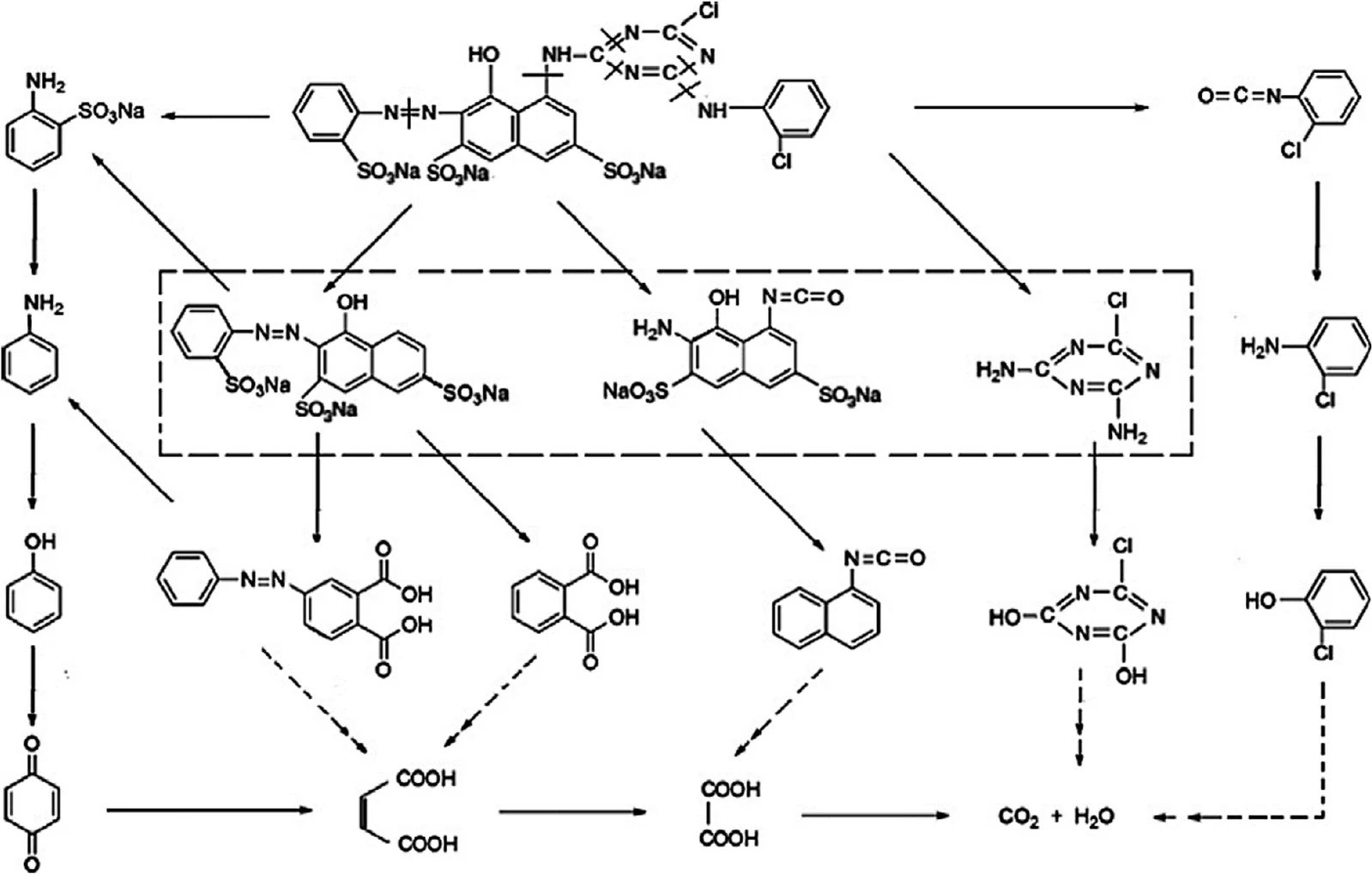
The possible degradation pathway of the dye K-2BP. The compounds in dashed frame may be possible intermediates. (Reprinted with permission from Ref. [76]. Copyright 2011 Elsevier).

On the other hand, interesting results have been reported from the viewpoint of analytical chemistry using similar experiments. Luo *et al*. [86] noticed that the sonochemical degradation rate of MO depends on the concentration of CCl_4_: a calibration curve was constructed to describe the relationship between the CCl_4_ concentration and the MO degradation rate (Fig. 6). Using this calibration curve, the CCl_4_ concentration was able to be determined by analyzing the change in the absorbance of aqueous MO-CCl_4_ solutions before and after sonication. Zhou *et al*. [28] also proposed a new method to determine the CCl_4_ concentration in water using ultrasound, where a calibration curve was constructed between the CCl_4_ concentration and the I_3_^−^ concentration formed by the Weissler reaction.

**Fig. 6:**
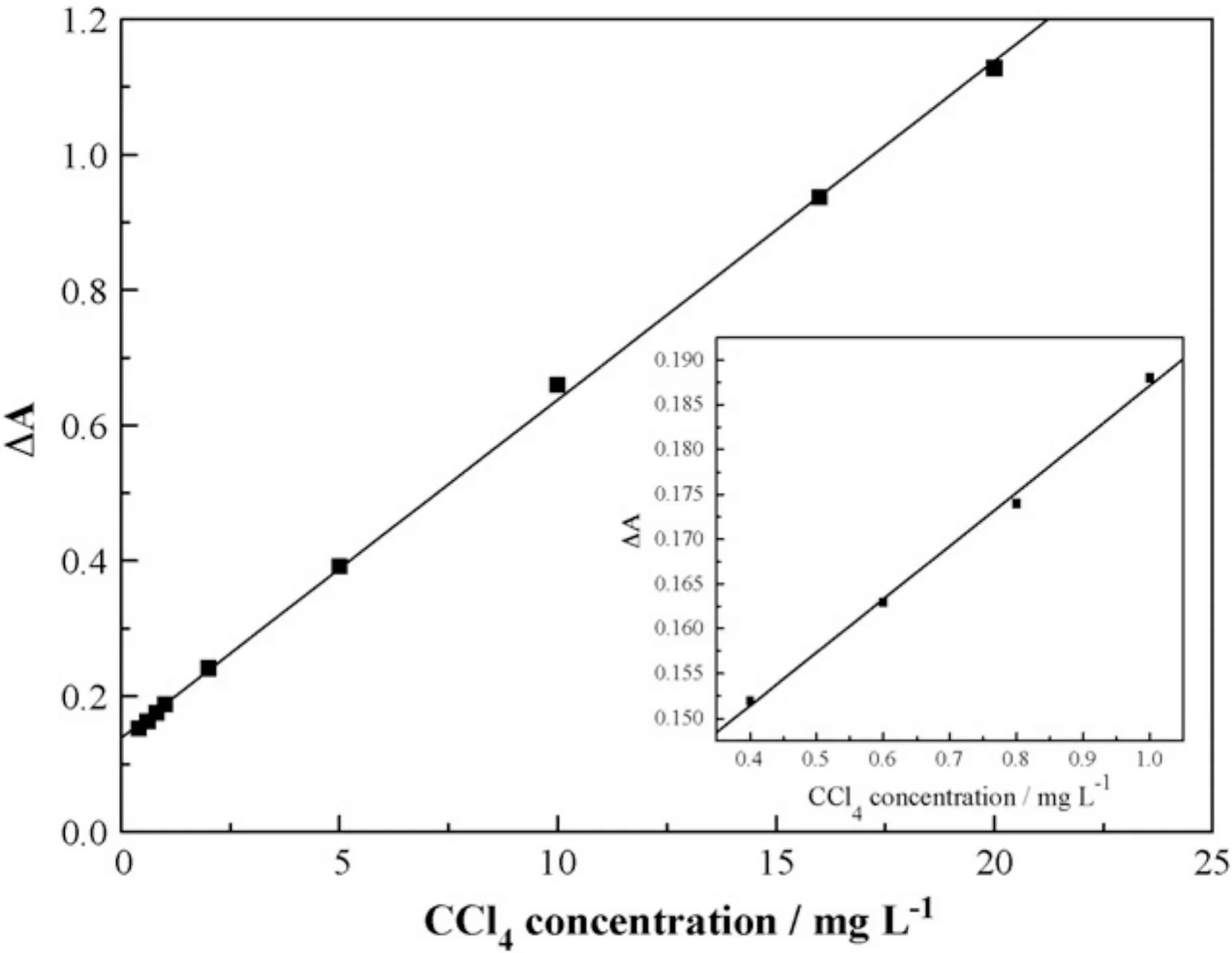
Working curve for CCl_4_ determination obtained under optimized reaction conditions. The initial MO concentration was 10 mg L^−1^, the initial solution pH was 2.0, and the sonication time was 12 min. The curve in the range of 0.4–1.0 mg L^−1^ is demonstrated by the inset [86]. Copyright, 2007 Elsevier.

## 5 Effect of CCl_4_ addition on degradation of aromatic compounds and antibiotics

The addition of CCl_4_ is also effective for the sonochemical degradation of aromatic compounds and antibiotics. Hua and Hoffmann reported that the degradation rate constant of nitrophenol increased 4.5 times by the addition of CCl_4_ [51]. Zheng *et al*. reported that the degradation rate constant of phenol increased 2.2–2.8 times by the addition of CCl_4_ [71], where CCl_4_ is reported to be a •H radical scavenger. Uddin and Okitsu reported that the degradation rate of various organic compounds was enhanced by the addition of CCl_4_ in the order of MO (9.6 times) > phenolic compounds (2.5–3.8 times) > methylene blue (1.4 times) [31]. In the degradation of phenolic compounds, the enhancement effect of CCl_4_ was smaller than that observed for MO. Table 5 shows that the addition of CCl_4_ was effective for the sonochemical degradation of phenols, levofloxacin, ciprofloxacin, cefadroxil, etc. [31], [51], [71], [88], [89], [90], [91], [92], [93], [94], [95].

The effect of CCl_4_ on the sonochemical degradation of four aromatic compounds was investigated in water [31]. In the absence of CCl_4_, the degradation rate followed the order of 4-chlorophenol > phenol > catechol = resorcinol. This order was in good agreement with the order of log *P*: 4-chlorophenol (log *P* 2.39) > phenol (log *P* 1.46) > catechol (log *P* 0.95) > resorcinol (log *P* 0.80). Organic compounds with higher log *P* values are more hydrophobic and thus they tend to degrade easier due to their higher concentration at the interfacial region of the bubbles [31], [94], [95]. On the other hand, in the presence of CCl_4_, a significant enhancement effect was observed: the enhancement effect of CCl_4_ on the degradation of aromatic compounds was 4-chlorophenol: 2.5 times, phenol: 3.7 times, catechol: 3.8 times, and resorcinol: 3.4 times, respectively. However, the rate of degradation of aromatic compounds in the presence of CCl_4_ did not follow the order of log *P*, suggesting that the effect of CCl_4_ on the rate of degradation would be dependent not only on the organic compound hydrophobicity but also on their chemical reactivity. In addition, Guateque-Londõno *et al*. reported that the good relationship between the hydrophobicity and degradation rate for, dicloxacillin (log *P* 1.93), MO (log *P* 0.20), and cefadroxil (CDX, log *P* − 1.73) in the absence of CCl_4_, where the ultrasonic irradiation was performed at 375 kHz and 990 kHz [94]. They discussed clearly the reaction sites by analyzing the degradation rates as a function of hydrophobicity. It was also observed that the addition of CCl_4_ can enhance the degradation rate of high hyhrophic compound of CDX.

Wei *et al*. [93] identified the degradation intermediates and products of ciprofloxacin by using an ultra-high-performance liquid chromatography system coupled with a Q-TOF mass spectrometer with electrospray ionization (ESI). The protonated molecular ion of ciprofloxacin ([M + H]⁺) was observed at *m*/*z* 332. They identified eight products as protonated molecular ions, where five products (P.1: *m*/*z* 304, P.2: *m*/*z* 306, P.5: *m*/*z* 348, P.7: *m*/*z* 362, and P.8: *m*/*z* 263) were related to the oxidation by •OH radicals. The progress of chlorine oxidation was also suggested by discussing three chloro-intermediates (P.3: *m*/*z* 322, P.4: *m*/*z* 340, P.6: *m*/*z* 366). They also investigated the effect of •OH radical scavenger, isopropanol, on degradation of ciprofloxacin. Based on eight observed intermediates and the results of the •OH radical scavenger experiments, the sonochemical degradation pathway of ciprofloxacin in the presence of CCl_4_ was suggested to be due to •OH radicals, HClO, Cl_2_, and chlorine-containing radicals (•Cl, •CCl_3_, and**:**CCl_2_). A proposed mechanism is shown in Fig. 7. However, the amounts of intermediates and products were not discussed in their paper. They also investigated agar diffusion tests with E. coli and S. aureus. The results showed that ciprofloxacin’s antibacterial activity was completely removed in 40 min. Although they did not discuss the effects of degradation products on antibacterial activity, it was suggested that the effect of ciprofloxacin on antibacterial activity was removed and the effects of degradation products on antibacterial activity were negligible to consider.

**Fig. 7:**
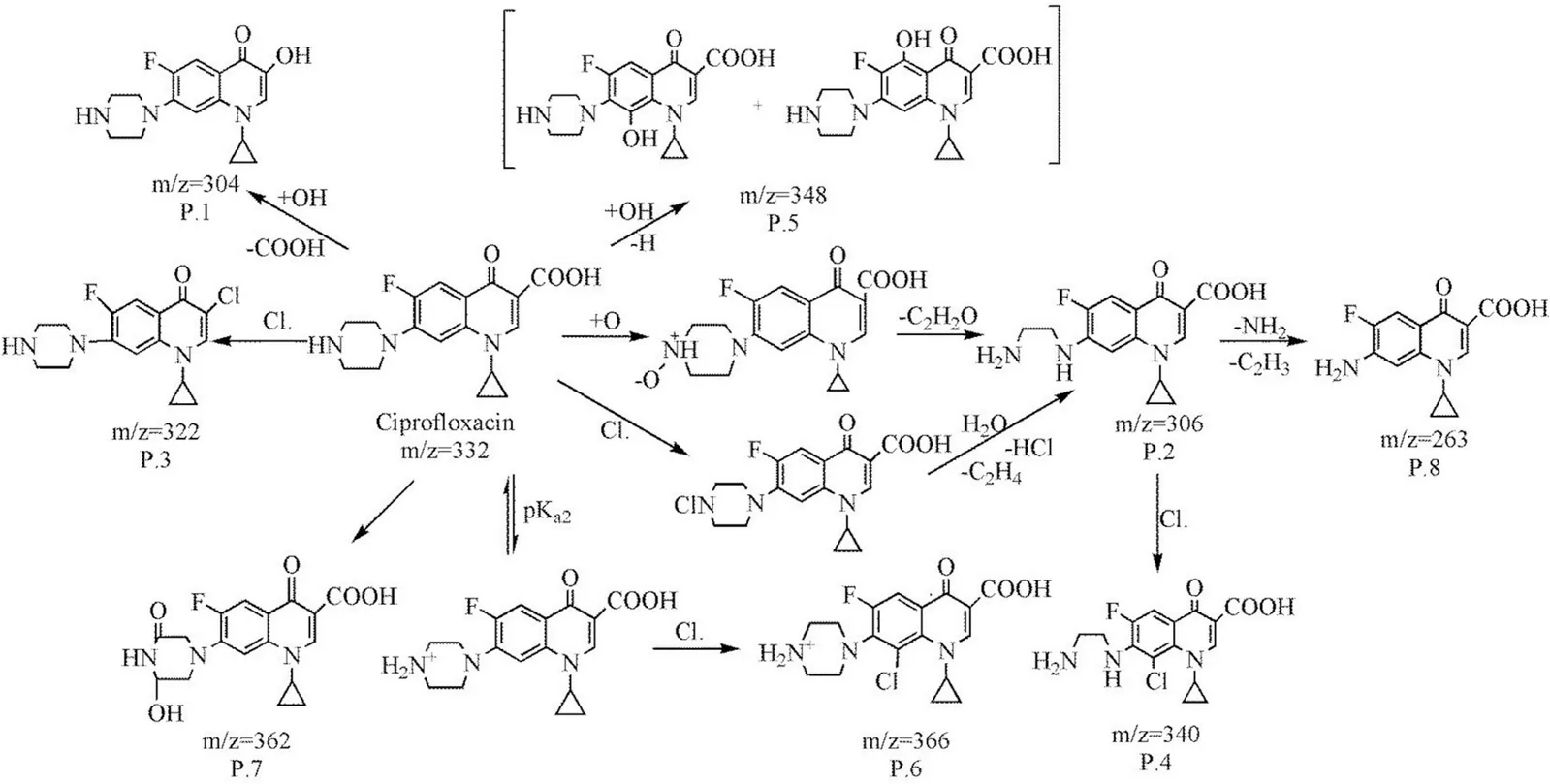
Proposed pathway for ciprofloxacin degradation under an ultrasound/CCl_4_ system. (Reprinted with permission from Ref. [93]. Copyright 2018 Elsevier).

When the sonochemical degradation of organic compounds was performed in the presence of CCl_4_, the chlorination of organic compounds could proceed as described in the degradation mechanisms of CCl_4_, K-2BP, and ciprofloxacin. It is well known that when natural water was disinfected by chlorination, toxic trihalomethane is formed [97], [98]. Since product analysis in the sonolysis of CCl_4_/organic compound systems is still limited, further investigation of the products and degradation mechanisms of organic compounds is needed as fundamental research. Recently, the reactivity of •Cl with an organic compound and the formation of chlorinated byproducts have been investigated [99], [100], [101]. Although the reactivity of •Cl is similar to that of •OH, the intrinsic difference in the reactions of •OH and •Cl with organic compounds has not been revealed [101]. Therefore, Qui et al. investigated the reaction mechanisms of •OH and •Cl with 13 aromatic and 11 aliphatic compounds by quantum chemical (QC) calculation and laser flash photolysis experiments [101]. To evaluate the relationship between the experimental and theoretical results, they monitored the change in the absorbance of the transient products (ex. radical cations) formed in the reactions of •OH and •Cl with organic compounds such as 1,3,5-trimethoxybenzen and bisphenol A. In their experiment, •OH and •Cl were generated by photolysis of 10 mM H_2_O_2_ and chloroacetone, respectively, under 266 nm UV irradiation. Some Cl-adducts were difficult to monitor the change in the absorption because of the overlap with the absorption of •Cl. In their paper, the reactions of radical adduct formation (RAF), single electron transfer (SET), and hydrogen atom transfer (HAT) were discussed. By analyzing the free energy of activation, they concluded that the oxidation of aromatic compounds mainly proceeds via RAF and SET by •OH and SET by •Cl, while the oxidation of saturated aliphatic compounds proceeds via only HAT by •OH and •Cl. Further experimental studies are need to clarify the reaction pathways of •OH and •Cl with organic compounds, because various reactions proceed in the degradation of organic compounds. In the case of an ultrasound/CCl_4_ system, since different amounts of •OH and •Cl are generated through complex processes during ultrasonic irradiation, the degradation mechanisms of organic compounds should be more complicated than those in the photolysis of H_2_O_2_ and chloroacetone. In addition, since target organic compound molecules are not homogeneously distributed among the three reaction sites (I, II, and III, as defined in the Introduction) in a sonicated solution containing numerous oscillating bubbles, its degradation rate is governed not only by its reaction rate constant but also by its physicochemical properties, such as vapor pressure and hydrophobicity/hydrophilicity [94], [95], [96]. The relationships between the behavior of organic compounds in a sonicated solution and their degradation rates and mechanisms should be investigated and discussed in future studies.

From the results of Table 2, Table 3, Table 4, Table 6, it can be summarized that the degradation of CCl_4_ proceeded faster than that of water, and the degradation of various hazardous organic compounds was accelerated in the presence of CCl_4_. These results are plotted in Fig. 8. The enhancement observed in Table 2, Table 4, Table 6 is due to the effect of the formed •Cl radicals and Cl-related oxidants, where the amount of •Cl radicals and Cl-related oxidants would be larger than that of •OH radicals formed. The clear enhancement effects were observed in Fig. 8. However, the degradation of organic compounds using CCl_4_ would have some problems. For example, incomplete degradation of CCl_4_ could lead to the hazard of the solutions, because CCl_4_ itself is toxic. In addition, if Cl-containing secondary compounds were formed, there would be concerns about their toxicity.

**Table 6:** Effects of CCl_4_ on sonochemical degradation of aromatic compounds (ACs) and antibiotics (As).

	Target chemical, Conc.	Sonicator type, Water temp.	Ultrasound frequency	Ultrasound power ^a)^	Dissolved gas, pH	Solution volume	CCl_4_ conc.^b)^	Rate of ACs and As degradation with CCl_4_^c)^	Enhancement	Notes (Rate without CCl_4_)
[51] Hua and Hoffmann (1996)	Nitorophenol 100 μM	Horn25℃	20 kHz	135 W	Ar	95 mL	Saturated solution (ca.5.2 mM)	(Not reported) Rate constant	4.5 times	Fig. 7
[71] Zheng *et al*. (2005)	Phenol 25 μM	Horn20℃	20 kHz pulsed mode (1 s on/1s off)	75–90 W acoustic power	air	50 mL	0.15 mM	0.0310 min^−1^ 0.061 min^−1^	2.2–2.8 times	(0.0138 min^−1^) (0.022 min^−1^) Table 1
[88] Mahamuni and Pandit (2006)	Phenol 70–92 ppm	Horn 31 ± 2℃	22 kHz	240 W	–	95 mL	413 ppm (ca. 2.7 mM)	0.0047 min^−1^	3 times	(0.0015 min^−1^) Table 1
[89] Lim *et al*. (2008)	2-chlorophenol 50 mg L^−1^	Bath 20 ± 1℃	20 kHz	250 W	air	250 mL	50 mg L^−1^ (0.325 mM)	(Not reported) Rate constant	2.4 times^d)^	Enhancement was calculated from the data points shown in Fig. 2
[90] Guo *et al*. (2010)	antibiotics levofloxacin 20 mg L^−1^	Horn 20 ± 1℃	20 kHz	200 W 17.3 W: cal.	air	50 mL	0.02 mL (4.15 mM)	(Not reported) time: 35min^e)^	7 times^e)^	Conclusion section
[91] Wang *et al*. (2012)	p-nitrophenol 0.1 mM	Horn 25 ± 1℃	20 kHz	200 W 14.5 W: cal.	air	50 mL	1500 mg L^−1^ (9.75 mM)	7.21 × 10^2^ min^−1^	27.7 times	(0.26 × 10^2^ min^−1^) Table 1
[92] Bagal and Gogate (2012)	alachlor, 20 ppm	horn 28 ± 2℃	20 kHz	100 W	air	100 mL	3 g L^−1^ (19.5 mM)	0.0471 min^−1^	3.1 times	(0.015 min^−1^) Table 3
[93] Son and Zoh (2012)	1,4-dioxane 78 μM	bath 5℃	170 kHz	15.4 W: cal.	Ar	(Reactor size: 2 L)	0.075 mM	(Not reported) time: 30 min	1.4 times^f)^	Enhancement was calculated from the data points shown in Fig. 7
[31] Uddin and Okitsu (2016)	4-chlorophenol phenol catechol resorcinol 150 μM	disk10℃	200 kHz	16 W: cal.	Ar	60 mL	2.6 mM	(Not reported) Initial rate	2.5times 3.7times 3.8times 3.4times	Fig. 8
[94] Wei *et al*. (2018)	ciprofloxacin, 20 mg L^−1^	Horn25-30℃	20–25 kHz	195 W	air	100 mL	41.4 mM	50.92 % Degradation ratio	100 times	(0.51 %) Fig. 1
[95] Guateque-Londoño *et al*. (2024)	Cefadroxil 30.6 μM	20 ± 2℃	375 kHz 990 kHz	94.08 W L^−1^: cal 79.4 W L^−1^: cal	–	250 mL	6.5 mM	(Not reported) time: 30 min	3.3 times5.4 times	Enhancement was calculated from the data points shown in Fig. 4

In Notes, the referred Table number, Figure number or section in the original paper are shown.

“—“: Not available.

a) Calorimetric power was abbreviated as cal. No description means nominal electric power.

b) Values in parentheses were converted to the unit of mM.

c) Yield at irradiation time (abbreviate as time), initial degradation rate (abbreviate as initial rate), or rate constant.

d) Data point at 120 min in Fig. 2 was used for rate estimation.

e) Data point at 35 min in Fig. 1 was used for rate estimation.

f) Data point at 30 min in Fig. 7 was used for rate estimation.

**Fig. 8:**
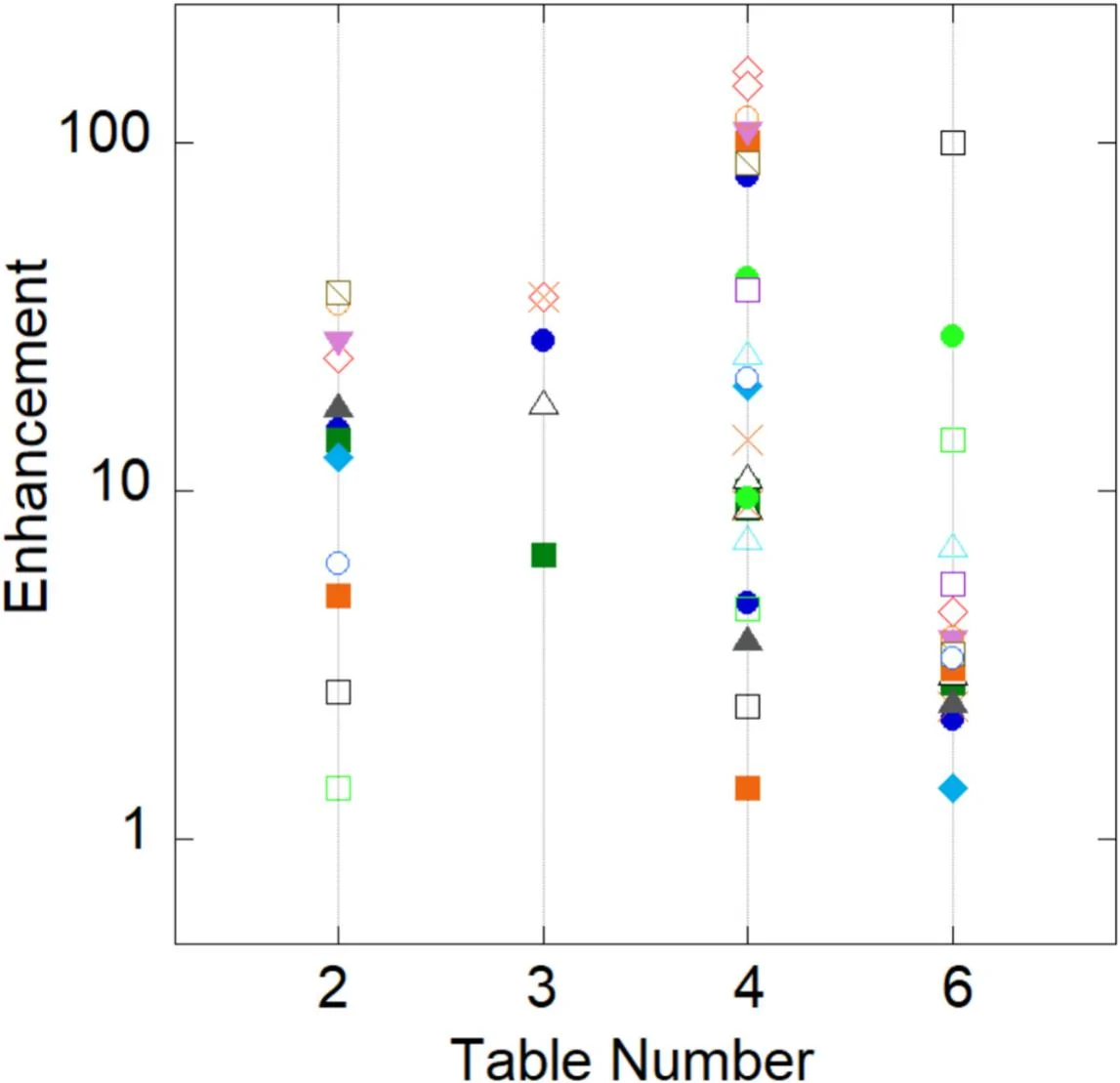
Enhancement summarized in Table 2 (enhancement for I^−^ oxidation), Table 4 (enhancement for dye degradation), and Table 6 (enhancement for organic compound degradation). Comparison the rate of CCl_4_ sonolysis with the rate of water sonolysis summarized in Table 3. These values are also shown as enhancement in Y-axis.

### 5.1 Environmental and practical considerations for CCl_4_ treatment

Here, the following aspects are discussed: (i) the acute and chronic toxicity of CCl_4_, (ii) the formation of chlorinated byproducts, (iii) the logistical challenges associated with handling and removing CCl_4_ from water, and (iv) the energy consumption of ultrasound system.

(i) Acute and chronic toxicity of CCl_4_

CCl_4_ is a highly toxic compound that causes severe liver and kidney damage upon acute exposure. Acute hepatic and renal complications have been reported in the cases of human poisoning [102], [103]. Experimental studies of CCl_4_ exposure have demonstrated marked hepatocellular degeneration and elevated serum transaminase activity. Furthermore, in the case of rats and mice, chronic exposure of CCl_4_ has been associated with liver fibrosis, cirrhosis, steatosis, and an increased incidence of hepatocellular tumors [104]. Therefore, when using CCl_4_ as a model pollutant or evaluating the significance of its sonochemical treatment, it will be necessary to take into account its high toxicity and potential carcinogenicity.

(ii) Formation of chlorinated byproducts

In the sonolysis of CCl_4_, it was reported that the formation of C_2_Cl_6_ (maximum concentration: about 80 nM) and C_2_Cl_4_ (maximum concentration: about 25 nM) was observed in the sonolysis of 0.20 mM of CCl_4_ at 25 min sonication [15]. After reaching their maximum concentrations, the concentrations of C_2_Cl_6_ and C_2_Cl_4_ decreased with increasing sonication time. This is in good agreement with the reports [105], [106], [107] where various volatile chlorinated compounds can be degraded by sonication. Furthermore, Drijvers *et al*. reported the results of the sonolysis of trichloroethylene (3.34 mM) by using 20 and 520 kHz ultrasound and analyzed the intermediates [108]. Volatile products identified by using GCMS and Kovats index were dichloroacetylene (C_2_Cl_2_), tetrachloroethylene (C_2_Cl_4_), tetrachlorobutenyne (C_4_Cl_4_), pentachlorobutadiene (C_4_HCl_5_), and hexachlorobutadiene (C_4_Cl_6_). The concentration of C_2_Cl_2_ increased to around 140 μM at 30 min sonication but this decreased to 20 μM at 80 min sonication under Ar. The concentrations of C_4_Cl_6_, C_4_Cl_4_, and C_4_HCl_5_ were less than 1 μM and the concentration of C_2_Cl_4_ was almost zero at 80 min sonication both under Ar and air. These results indicated that volatile intermediates and products were further degraded as sonication continued. As mentioned above, in the sonolysis of K-2BP and ciprofloxacin in the presence of CCl_4_, several chloro-intermediates and products were detected, but those amounts were unclear. The characterization of intermediates and products formed during sonolysis, as well as the assessment of their toxicity, remains limited to date.

For comparison, the byproducts formed by chlorination in the absence of ultrasound are briefly introduced. It has been reported that chlorination of natural water could result in the formation of toxic trihalomethanes [97], [98] and that phenolic compounds in water could react with free chlorine during chlorination to form chlorophenols [109], [110]. Onodera *et al*. reported that various chlorinated phenols were formed through the chlorination of phenol; however, these phenols were subsequently degraded into organic acids or humic acid-type compounds at pH 7. [109]. In future studies, the potential formation of chlorinated byproducts during sonochemical degradation of organic compounds in the presence of CCl_4_ should be investigated, and their degradation mechanism and toxicity should be assessed alongside treatment performance.

(iii) Logistical challenges of handling and removing CCl_4_ from water

In Japan, the environmental quality standard for CCl_4_ in water is 0.002 mg/L, while the effluent standard is 0.02 mg/L [111], which is ten times the environmental quality standard and corresponds to approximately 0.13 μM. As summarized in Table 3, the sonochemical degradation of CCl_4_ have mainly investigated at relatively high concentrations (6.5 μM–3.3 mM) using solution volumes of 65–2000 mL. For example, Nagata et al. [49] investigated the sonochemical degradation of CCl_4_ at a concentration of 3.3 mM in a 65 mL solution. This CCl_4_ concentration is approximately 25,000 times higher than the effluent standard. The differences in concentration and treatment volume would indicate the logistical challenges associated with applying sonochemical treatment to CCl_4_-contaminated water in practical situations. In particular, CCl_4_ must be removed to very low concentrations while treating a sufficiently large volume of water. In addition, the high volatility of CCl_4_ requires careful handling and prevention of volatilization during treatment and sampling. Because CCl_4_ is not readily degraded by •OH radicals, the available methods for degrading CCl_4_ in aqueous systems are limited. One approach to mitigating CCl_4_ contamination is to transfer CCl_4_ from the aqueous phase to the solid phase or gas phase rather than degrade it, using methods such as activated carbon adsorption [112], [113] and air stripping [114], [115]. However, these methods merely remove CCl_4_ from the water and do not destroy CCl_4_ itself; therefore, further treatment is required to degrade the removed CCl_4_. In recent years, reductive degradation of CCl_4_, as discussed in a later section, has been actively investigated.

(iv) Energy consumption of ultrasound system

The energy consumption of the ultrasound system for the sonolysis of CCl_4_ in water was evaluated. In the case of a high intensity horn type sonicator of 20 kHz, the strong physical effects of cavitation may induce the transfer of CCl_4_ from the aqueous phase to the gas phase, suggesting that the rate constants determined from the residual CCl_4_ concentration in water may be subject to experimental errors. Therefore, we used the results of Hung amd Hoffmann [15], where the first-order rate constant for CCl_4_ degradation was obtained by the use of a disk type sonicator of 500 kHz. The following data and experimental conditions were used for calculation: a rate constant of 0.070 min^−1^ and initial CCl_4_ concentration of 0.2 mM in a solution volume of 483 mL. The calorimetric power was 35 W, whereas the electrical power was assumed to be 100 W.

The energy consumption of the ultrasound system was calculated by using the equations proposed by Bolton et al. [116]. They defined the electric energy per order of magnitude (E_EO_, unit: kWh m^−3^/order) for situations in which an organic pollutant is present at a low concentration and the reaction follows first-order kinetics. In this calculation, the following definition was also done: E_EO_ is the electric energy in kilowatt hours [kWh] required to degrade an organic pollutant by one order of magnitude in a unit volume [eg., 1 m^3^ (1000 L)] of contaminated water. In other words, the calculation was performed to estimate the energy required for 90% removal of the organic pollutant.

In the case of a batch reactor, the following equation is reported [116];

(31) $$E_{\mathrm{EO}}=Pt\text{1}000/V\text{lg}\;\left(c_{\text{i}}/c_{\text{f}}\right)$$

where *P* is the rated power [kW], *t* is the treated time [h], *V* is the volume [L], lg is the symbol for the common logarithm, and *c*_i_ and *c*_f_ are the initial and final concentrations [mol L^−1^, M] of pollutant. Since lg(*c_i_*/*c_f_*) is equal to 0.4343 *k’t*, where *k’* is the first-order rate constant [min^−1^] and *t* is the reaction time [min], Eq. (31) becomes

(32) $$E_{\mathrm{EO}}=\;\text{38}.\text{4}P/Vk^{\prime }$$

where *V* is batch reactor volume [L]. By using *P* of 0.100 kW, *V* of 0.483 L, and *k*’ of 0.070 min^−1^, *E*_EO_ can be calculated 114 kWh m^−3^/order. This value was considerably higher than those reported for other AOPs. For example, Sgroi *et al*. calculated the energy consumption for AOPs operated at pilot-scale [117]. *E_EO_* values of ozone based AOPs were less than 0.6 kWh m^−3^/order and *E_EO_* values of H_2_O_2_ based AOPs were less than 15 kWh m^−3^/order, where these values were calculated with batch operation or flow-through operation for quite low concentrations (21–1813 ng L^−1^) of emerging organic pollutants such as carbamazepine fluoxetine, gemfibrozil, primidone, sulfamethoxazole, trimethoprim. An accurate comparison between the ultrasound system and other AOPs is difficult because CCl_4_ is generally not degraded by O_3_/UV and H_2_O_2_/UV and because of differences in pollutant concentrations, experimental scales, and treatment configurations, such as batch and flow systems. However, it is clear that the ultrasound system calculated here currently requires relatively high electrical energy consumption. Therefore, the practical applicability of the ultrasound system to low-concentration CCl_4_-contaminated water should be further investigated, particularly in terms of its energy efficiency and scalability. Although the ultrasound system has demonstrated the potential to degrade CCl_4_, its relatively high electrical energy consumption at the laboratory scale may limit its practical application to water containing environmentally relevant concentrations of CCl_4_. Future studies should therefore evaluate the treatment performance and energy efficiency at lower CCl_4_ concentrations and larger treatment scales, as well as assess the feasibility of scaling up the process from batch systems to continuous-flow systems.

It is important to consider plausible alternatives based on the underlying mechanism. For example, the addition of halocarbons with higher vapor pressures than CCl_4_ but lower toxicity may be considered as a potential approach to enhancing the degradation of organic compounds. However, the degradation rates of CHCl_3_ and CH_2_Cl_2_ were lower than that of CCl_4_, indicating that vapor pressure does not necessarily correlate directly with the sonochemical degradation rate [15]. This point is discussed in the results shown in Fig. 2. On the other hand, the effect of chloral hydrate (CCl_3_CH(OH)_2_) on the Weissler reaction, which is less toxic than CCl_4_, has been investigated as an alternative of CCl_4_ [118]. However, the concentrations of I_3_^⁻^ formed during the sonolysis of chloral hydrate were very low, being only approximately 11–15% of those formed in the presence of CCl_4_. Therefore, chloral hydrate was not a suitable alternative to CCl_4_ for enhancing the degradation of organic compounds.

At present, no halocarbons have been identified as suitable alternatives to CCl_4_. However, non-halogenated compounds such as radical precursors may be used as alternatives to enhance the degradation of organic compounds without the addition of CCl_4_. For example, sulfate radicals generated through the activation of persulfate have been extensively studied for water treatment. Various activation methods have been investigated for persulfate, among which ultrasonic activation can generate sulfate radicals [119], [120]. Choi *et al.* recently reviewed the use of sulfate radicals generated through ultrasonic activation for the effective degradation of hazardous organic compounds [120]. However, in such cases, the target compounds are generally limited to those that can be oxidized by sulfate radicals and •OH radicals.

In addition, the combination of ultrasound system with photocatalysts has been actively studied to enhance the rate of degradation and to reduce the amount of hazardous intermediates. For example, an OH⁻-regulated SrBi_4_Ti_4_O_15_/BiOCl heterojunction sonophotocatalyst has been reported to effectively degrade tetracycline, an antibiotic, while reducing the toxicity of the treated solution compared with that of the original tetracycline solution [121]. Yu *et al*. proposed that interfacial electron transfer involving metal–chloride interactions contributed to the enhanced sonophotocatalytic activity. These findings provide new insights into the design of efficient sonophotocatalytic systems based on metal–chloride interfacial electron transfer.

We also consider the combination of the ultrasound/CCl_4_ technique with the reductive dechlorination technique described in Section 6 to be a promising approach. The reductive dechlorination technique is discussed in the following section.

## 6 Reductive degradation of CCl_4_

In recent years, reduction reactions have attracted considerable attention in the degradation of organohalogen compounds. For example, per- and polyfluoroalkyl substances [122], trichloroethylene [123], polychlorinated biphenyls [124] and polybrominated diphenyl ethers [125] can be degraded by using various reduction techniques. It has been reported that an electron transfer reaction occurs between the surface of metal powders and organohalogen compounds, and thus the reductive degradation of the compounds can proceed [126], [127].

In the ultrasonic method, it has been reported that CCl_4_ can be converted to CH_4_ by adding transition metal powders to an aqueous solution containing CCl_4_ [128], [129], because shock waves generated by ultrasonic cavitation are used to obtain a clean and reactive metal surface on the metal powders. Hung *et al*. reported that the reductive degradation of CCl_4_ and nitrobenzene was promoted by ultrasonic irradiation with Fe powder [128], [130]. This is because the clean metal surface, treated by the shock waves of ultrasonic cavitation, acts as an effective reaction site. This idea is supported by the mechanism proposed by Suslick and Doktycz. They reported that the Reformatsky reaction of benzaldehyde with ethyl bromoacetate to yield ethyl-3-hydroxy-3-phenylpropionate was remarkably promoted on the existence of clean surface of Zn [131]. This is because the inactive ZnO layer on the Zn powder was removed by ultrasonic irradiation. Okitsu *et al*. reported the sonochemical degradation of CCl_4_ in an aqueous solution containing transition metal powders of Zn, Fe, and Cu [129]. The degradation rate was in the order of no metal powder < Cu < Fe < Zn. The addition of Zn powders accelerated the rate of degradation of CCl_4_ by about 33 times compared with that in the absence of Zn powders. One reason why the degradation rate of CCl_4_ differed among Cu, Fe, and Zn may be their different redox potentials (Cu^2+^ + 2e → Cu; 0.337 V, Fe^2+^ + 2e → Fe; −0.440 V, Zn^2+^ + 2e → Zn; −0.7628 V) [129]. In the reaction between Zn metal and CCl_4_, it has been reported that the reductive dechlorination reaction would proceed as shown in Eq. (33) even in the absence of ultrasound [127].

(33) 2Zn(s) + 2CCl_4_ + 2H_2_O → ZnCl_2_(aq) + Zn(OH)_2_(s) + 2CHCl_3_

where (s) denotes the condition of solid. Here, CCl_4_ is reduced by accepting electrons from Zn and chlorine is released as chloride ions: Zn acts as a reducing agent donating electrons.

In order to proceed the reductive dechlorination reactions efficiently, it is necessary to increase the number of active sites on the surface of metal powders which have moderate reducing ability. In general, shock waves can be easily generated using a conventional ultrasonic cleaning bath, which has a low initial cost. Furthermore, since this method involves the irradiation of low-energy ultrasound, it also offers the advantage of keeping the running cost low. From an energy conservation perspective, it is important to develop new water treatment technologies using shock waves in the future.

## 7 Conclusion

The fundamental reactions involved in the sonolysis of CCl_4_ in water were reviewed from the perspectives of I^–^ oxidation in the presence of CCl_4_, CCl_4_ degradation, degradation of hazardous organic compounds in the presence of CCl_4_, and reductive degradation of CCl_4_. The chemical reactions induced by high-temperature bubbles, radicals, and activated metals were discussed to provide a better understanding of ultrasound-based water treatment. The I^−^ oxidation reaction was accelerated 1.4–37 times by the addition of CCl_4_. The Weissler reaction would be useful for evaluating the chemical effects of a low-power ultrasound apparatus or relatively low-temperature bubbles that are unable to degrade water. It was also confirmed that the rate of degradation of CCl_4_ was 3.3–36 times higher than that of water. Furthermore, by the addition of CCl_4_ in the sample aqueous solution, the rate of degradation of dyes was accelerated 1.4–160 times, and the rate of degradation of aromatics and antibiotics was accelerated 1.4–100 times. These results were attributed to the low binding energy of the Cl–CCl_3_ bond compared with other chemical bonds and the moderate accumulation behavior of CCl_4_ towards the inside and the interfacial region of bubbles. Because the addition of CCl_4_ accelerated the degradation of various hazardous organic compounds, it is expected that CCl_4_ could accelerate the degradation of the organic compounds that have not been investigated so far. The reductive degradation was suggested to occur in the degradation of chlorinated organic compounds. Therefore, the research on the combination of sonochemical degradation and reduction reaction may be also effective to accelerate the degradation rate. This combination may be also useful to reduce the amounts of Cl-containing compounds. In the future, it will be important to further improve the efficiency of CCl_4_ degradation and to search for alternative organic compounds to CCl_4_. It is also needed to clarify the relationship between the bubble temperature and degradation characteristics, as well as the behavior of bubbles and organic molecules in the solution. In addition, the number and size of bubbles reaching high temperatures and pressures, as well as the average temperature and pressure at which the reactions occur, should be clarified in more details. Few studies have reported on the degradation mechanism in the presence of CCl_4_. It remains unclear to what extent chlorine-containing compounds are present as intermediates. Further elucidation of the degradation mechanism and assessment of the potential hazards of the treated water are important challenges for future research.

## CRediT authorship contribution statement

**Kenji Okitsu:** Writing – review & editing, Writing – original draft, Investigation, Funding acquisition. **Aerfate Abulikemu:** Writing – review & editing, Writing – original draft, Investigation.

## Declaration of competing interest

The authors declare that they have no known competing financial interests or personal relationships that could have appeared to influence the work reported in this paper.
